# A synthesis of 1β-hydroxytestosterone, a metabolite of xenobiotic human cytochrome P450 enzymes, beginning with a borylation of boldione

**DOI:** 10.1039/d5ob01218j

**Published:** 2025-09-09

**Authors:** Anna I. Elizondo, Kevin D. McCarty, Hadi D. Arman, F. Peter Guengerich, Francis K. Yoshimoto

**Affiliations:** a Department of Chemistry, the University of Texas at San Antonio (UTSA) San Antonio Texas 78249 USA francis.yoshimoto@gmail.com; b Department of Biochemistry, Vanderbilt University School of Medicine Nashville Tennessee 37232-0146 USA

## Abstract

Xenobiotic cytochrome P450 enzymes have been shown to hydroxylate testosterone at various positions in the steroid backbone, including C1 to produce 1β-hydroxytestosterone. Despite the potential application to study the biochemistry of these enzymes, 1β-hydroxytestosterone is not commercially available. A synthesis of 1β-hydroxytestosterone from commercially available boldione (androst-1,4-dien-3,17-dione) was accomplished in eight steps. The key step to functionalize C1 was a borylation reaction catalyzed by an *in situ* generated copper carbene complex. The synthetic strategy reported will be used to access other biologically relevant C1-hydroxylated steroids to explore the biochemistry of drug metabolizing P450 enzymes.

## Abstract 2

An 8-step synthesis of 1β-hydroxytestosterone was accomplished beginning with a conjugate borylation reaction of boldione.

## Graphical abstract

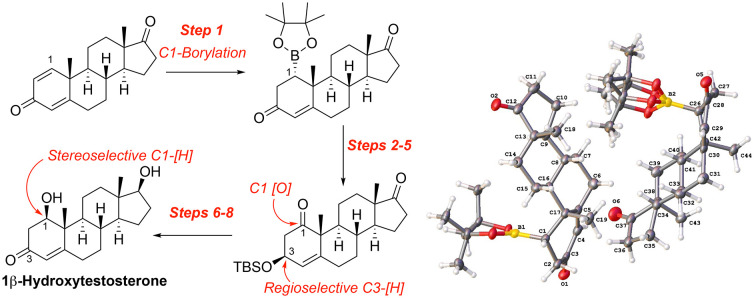

## Background

**Fig. 1:**
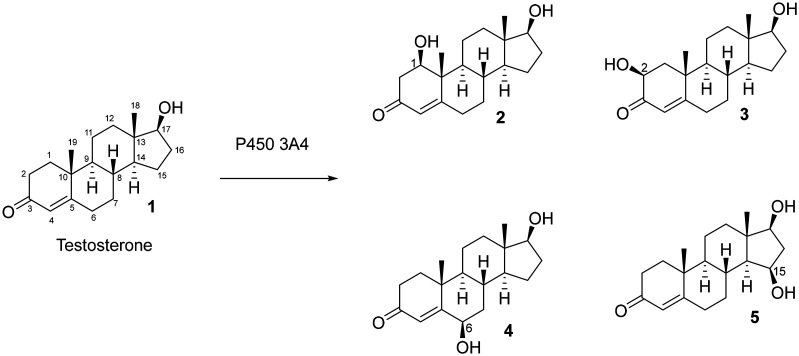
Testosterone (1) hydroxylation catalyzed by cytochrome P450 3A4 yields monohydroxylated products at C1, C2, C6, and C15 (2, 3, 4, and 5).^3^

Testosterone has been used as a substrate to biochemically characterize human xenobiotic P450 enzymes.^1,2^ For instance, cytochrome P450 3A4, which is a drug metabolizing liver enzyme, incorporates a hydroxy group at the 1β-, 2β-, 6β-, and 15β-positions of testosterone – the product distribution is in a ratio of 6.7 : 13 : 73 : 6.7 based on the reported *k*_cat_ values, respectively (Fig. 1).^3^ In contrast, P450 3A7, which is overexpressed in fetal liver, has been shown to monohydroxylate testosterone with a different regioselectivity.^4^ Due to its potential application to study xenobiotic drug metabolizing P450 enzymes, authentic standards of hydroxylated testosterone derivatives would be useful to study their biochemistry. However, enzymatic conversion could be low yielding^5^ and restricted to specialized laboratory equipment and plasmid strains,^6^ which directed our focus to accessing the compound through chemical synthesis.

The prior reports for the syntheses of 1β-hydroxytestosterone^7^ and 1α-hydroxytestosterone^8^ both involved 7 steps from 5α-dihydrotestosterone benzoate with yields of 8.5% and 1.8%, respectively. The incorporation of the 1-oxygen was obtained from the epoxidation using *t*-butylhydroperoxide in the presence of molybdenum hexacarbonyl or NaOH to eventually yield the 1β-hydroxy or the 1α-hydroxy derivatives, respectively. Our research laboratory previously reported the direct C–H hydroxylation at C1 using the Schönecker oxidation conditions,^9^ but this method would be restricted to the 5α-reduced and 19-oxo steroid backbone.

**Fig. 2:**
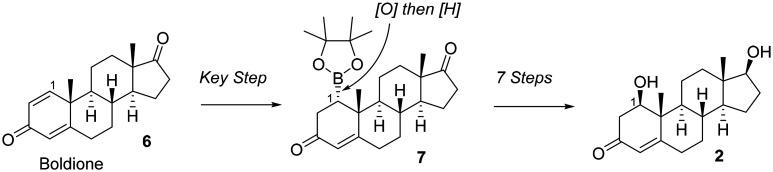
Overall strategy in this report to synthesize 1β-hydroxytestosterone (2) from boldione (androst-1,4-dien-3,17-dione, 6) through a borylated intermediate (7).

Here, we report the synthesis of 1β-hydroxytestosterone (2) beginning with a key 1,4-borylation at C1 onto commercially available boldione (androst-1,4-dien-3,17-dione) (Fig. 2, 6 to 7 to 2). The synthesis of 1β-hydroxytestosterone (2) from boldione (6) was achieved in eight total steps.

## Results and discussion

### Optimization of the 1,4-borylation reaction onto androst-1,4-dien-3,17-dione using DBU

**Scheme 1:**
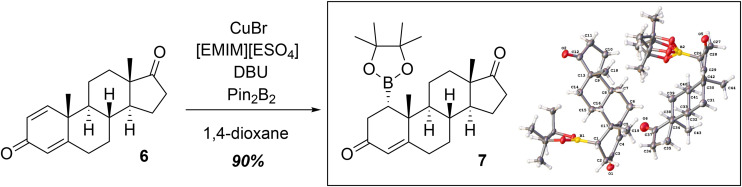
Successful 1,4-borylation of androst-1,4-dien-3,17-dione (6) to yield borane adduct 7. [EMIM][ESO_4_]: 1-ethyl-3-methylimidazolium ethyl sulfate (Step 1 of 8 steps).

Although others have reported the conjugate addition of pinacolatoborane onto α,β-unsaturated carbonyls,^10,11^ our efforts to replicate the various reaction conditions onto androst-1,4-dien-3,17-dione resulted in either no reaction or low yield^12^ with the copper–carbene system. We hypothesized that the low yield of the copper-catalyzed reaction was due to the base (potassium *tert*-butoxide), which made the reaction mixture viscous and cloudy with the presence of precipitate when stirring. Therefore, the base was switched to 1,8-diazabicyclo-(5.4.0)-undec-7-ene (DBU), which resulted in a homogeneous mixture. Table 1 summarizes the optimization conditions (see Fig. S2-2 for the NMR spectroscopic overlay of the 5 entries). Unlike a prior report,^13^ the presence of copper was required in our system for the reaction to occur. The use of DBU as the base optimized the yield of the C1-borylated product to 90% (Scheme 1, 6 to 7).

**Table 1:** Optimization of Step 1: C1-borylation of boldione (6) to yield 7

Entry	Conditions	Yield^a^
1	(PinB)_2_ (1.0 eq.), PPh_3_ (0.76 eq.), CH_3_OH, Wilkinson's Catalyst (0.3 eq.)	18%
2	(PinB)_2_ (1.1 eq.), THF, DBU (45 eq.), [EMIM][ESO_4_] (45 eq.)	—b
3	(PinB)_2_ (3.5 eq.), THF, KO*t*Bu (7.1 eq.), CuBr (9.3 eq.), [EMIM][ESO_4_] (4.2 eq.)	39%
4	(PinB)_2_ (1.3 eq.), THF, DBU (1.8 eq.), CuBr (0.2 eq.), [EMIM][ESO_4_] (1.8 eq.)	75%
5	(PinB)_2_ (1.5 eq.), 1,4-D,^c^ DBU (1.0 eq.), CuBr (0.2 eq.), [EMIM][ESO_4_] (1.0 eq.)	88%

a Yield of 7 was calculated by integration of the C4-protons of 6 and 7 of the ^1^H NMR spectra of the crude reaction mixtures (*δ* 6.3 and 5.8, respectively. Also see Fig. S2-2).

b No C4-vinyl proton corresponding to 7 in the crude reaction mixture was detected by ^1^H NMR spectroscopy.

c (1,4-D): 1,4-dioxane.

Similar to other reports of the borylation at C1 of the steroid, the boron substituent was added in the α-orientation, presumably to avoid the steric clash with the C19-axial methyl of the starting material. A crystal structure of the boron adduct is shown in Scheme 1, which confirms the stereochemistry at C1. To test the versatility of the borylation reaction, androst-2,4-dien-1-one was also used as the substrate, which underwent conjugate addition at C3 (see SI).

### A 2-step sequence to 1α-hydroxytestosterone from C1–borane adduct 7

**Scheme 2:**
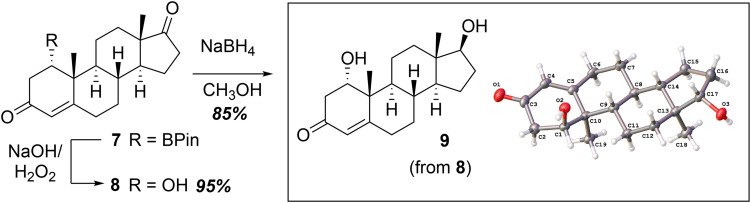
Synthesis of 1α-hydroxytestosterone (9) from C1-borylated steroid intermediate (7).

Treatment of borane 7 with stoichiometric H_2_O_2_ and NaOH in THF gave 1α-hydroxyandrostenedione (Scheme 2, 8, Step 2 of 8 steps). This oxidation of the C1–boryl substituent to the alcohol was stereospecific and retained the C1α-orientation of the substituent (*i.e.* the 1α-borane substituent was oxidized to the 1α-hydroxy substituent with H_2_O_2_). Reduction of the C17-ketone of 1α-hydroxyandrostenedione with NaBH_4_ in CH_3_OH gave 1α-hydroxytestosterone (9). This route to 1α-hydroxytestosterone from the boron intermediate contrasts with the previous strategy^7,8^ to incorporate the 1-hydroxy group, which involved the nucleophilic epoxidation of the 3-keto-Δ^1^ steroid followed by reduction with LiAlH_4_ to yield the C1α-hydroxy steroid.

### Conversion of 1α-hydroxyandrostenedione to 1β-hydroxytestosterone (8 to 2)

**Scheme 3:**
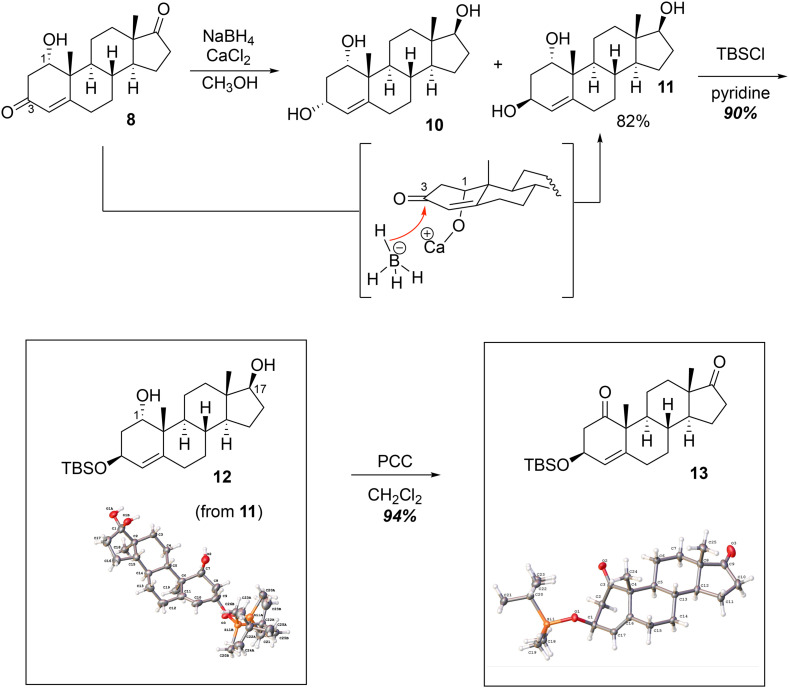
Synthesis of 3-*tert*-butyldimethylsiloxy-1,17-diketo-androst-4-ene (13) from 1α-hydroxyandrostenedione (8) (Steps 3–5 of 8 steps). The intermediate in brackets explains the stereoselective reduction at C3 through the chelation of the calcium Lewis acid with the C1α-hydroxy group and the borohydride to deliver the hydride at the bottom face, yielding 11.

For the synthesis of 1β-hydroxytestosterone (2), various methods were performed to incorporate the 1β-hydroxy group (see section S12) but the ultimate strategy involved the oxidation of the 1α-hydroxy group to the C1-ketone, which was stereoselectively reduced to the 1β-hydroxy group (Scheme 3). To begin, 1α-hydroxyandrostenedione was treated with CaCl_2_ and NaBH_4_ in CH_3_OH^14^ to primarily afford the 3β-hydroxy epimer 11 in 82% yield with a minor amount of the 3α-hydroxy epimer 10 (Scheme 3, 2 to 11, Step 3 of 8 steps). The presence of the CaCl_2_ forms Ca(BH_4_)_2_, which in turn enables calcium to chelate^15,16^ with the C1-hydroxy group of the substrate and the borohydride reducing agent. This chelation directs the hydride to attack on the bottom face giving the 3β-hydroxy epimer (11) as the major product. Alternatively, the Luche reduction conditions (NaBH_4_ in the presence of CeCl_3_·7H_2_O) yielded an epimeric mixture of the triols 10 and 11 (45 : 55 ratio, see SI for details) due to the lack of a chelation with the C1–oxygen. Triol 11 was regioselectively protected at C3 as the TBS ether 12 using TBSCl and pyridine as both the base and the solvent (11 to 12, Step 4 of 8 steps). Pyridine was required to dissolve the triol (11). The resulting 1,17-diol (12) was oxidized with 3 mol eq. of PCC in CH_2_Cl_2_ to yield the diketone 13 (12 to 13, Step 5 of 8 steps), which was used as the precursor to introduce the key 1β-hydroxy group in the steroid backbone.

The stereoselective reduction of the C1-ketone intermediate (13) to yield the 1β-hydroxy epimer was not trivial. Our past work in the stereoselective reduction of a C12-ketone guided us in this optimization process.^17^Table 2 shows a set of reaction conditions, which led us to conclude that the Luche reduction at −78 °C was the optimal method to yield the desired 1β-hydroxy stereoisomer. The use of l-selectride as a sterically hindered hydride source (Table 2, entry 1), gave mostly the 1α-hydroxy epimer product (12) (74: 26, 12 to 14). On the other hand, a smaller reducing agent such as NaBH_4_ gave more of the desired 1β-hydroxy epimer product (14) relative to l-selectride (entry 2, 54: 46, 12 to 14). In addition, three factors to optimize this reaction followed (see entry 3): (i) lowering the temperature to −78 °C, (ii) the use of THF as a co-solvent to enhance solubility of the C1-ketone starting material (13), and (iii) the addition of CeCl_3_·7H_2_O to ensure reactivity of the hydride at −78 °C. The successful stereoselective reduction of 1,17-diketone 13 using CeCl_3_·7H_2_O and NaBH_4_ in CH_3_OH and THF at −78 °C gave the desired 1β,17β-diol 14 (Table 2, entry 3, 6.0 : 94, 12 to 14, Step 6 of 8 steps). When the reaction was performed at rt, the C1-epimers (1α/1β hydroxy epimers) were obtained in a 1 to 1 ratio (entry 4). The low temperature of the reduction with a small reducing agent (Luche conditions) avoids torsional strain between C9 and the oxygen (see Fig. S3-3).

**Table 2:** Optimization of Step 6: stereoselective reduction of C1-ketone (13) to yield primarily the 1β-hydroxy epimer

Entry	Reaction conditions	1α-Hydroxy (12)	1β-Hydroxy (14)
1^a^	l-Selectride, THF, −78 °C	74%	26%
2^a^	NaBH_4_, CH_3_OH, rt	54%	46%
3^a^^,^^c^	NaBH_4_, CeCl_3_·7H_2_O, CH_3_OH/THF, −78 °C	6.0%	94%
4^b^^,^^d^	NaBH_4_, CeCl_3_·7H_2_O, CH_3_OH/THF, rt	47%	53%

a The ratio of the α- and β-hydroxy epimers (12 and 14) were determined by integrating the Δ^4^ proton at *δ* 5.32 and 5.27.

b The ratio of the α- and β-hydroxy epimers (12 and 14) were determined by TLC analysis *R*_f_: 0.634 and 0.846, respectively (1 to 1 ethyl acetate/hexanes, v/v).

c 1 to 1 ratio of CH_3_OH/THF, 2 mol eq. of NaBH_4_, 2 mol eq. of CeCl_3_·7H_2_O.

d 1 to 1 ratio of CH_3_OH/THF, 3 mol eq. of NaBH_4_, 2 mol eq. of CeCl_3_·7H_2_O.

Deprotection of the 3-TBS group with excess TBAF in THF afforded triol 15 (Step 7 of 8 steps). Triol 15 was regioselectively oxidized at C3 with PDC (1 mol equivalent) in pyridine to furnish 1β-hydroxytestosterone (2) in 80% isolated yield (Step 8 of 8 steps). When pyridine was used as the solvent, the triol was completely soluble and the main product isolated was the desired oxidation product at C3 to yield 1β-hydroxytestosterone (2). The C3-hydroxy group is the least sterically hindered alcohol among the three positions of triol 15 (*i.e.* C1, C3, and C17) where C1 and C17 are both adjacent to a quaternary carbon center (C10 and C13, respectively). The regioselective oxidation of the less hindered C3 alcohol over the more congested alcohols at C1 and C17 is reminiscent of a prior study, which used cholesterol oxidase^18^ to selectively oxidize the C3-position of 7α-hydroxycholesterol to yield 7α-hydroxy-cholest-4-en-3-one.

## Conclusion

**Scheme 4:**
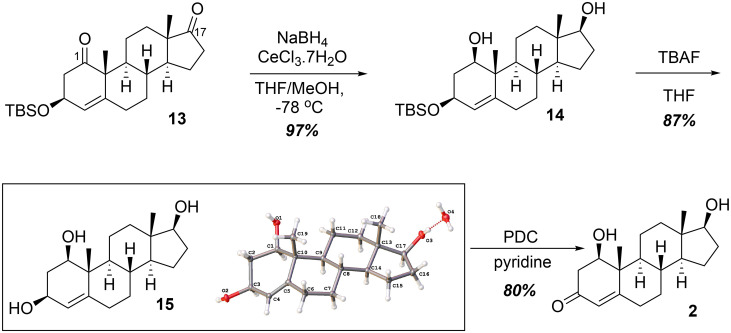
Synthesis of 1β-hydroxytestosterone (2) from 1,17-diketone 13 (Steps 6–8 of 8 steps).

In conclusion, a synthesis of 1β-hydroxytestosterone (2) was achieved from commercially available androst-1,4-dien-3,17-dione (6) through a 1,4-borylation reaction (Scheme 1). The use of DBU as the base to generate the carbene was necessary to optimize the yield for the conjugate borylation (see Fig. S2-2). Other key steps include: (i) the regioselective and stereoselective reduction of a 3-keto-Δ^4^-intermediate to yield a 3β-hydroxy Δ^4^ product using CaCl_2_ and NaBH_4_ in CH_3_OH^14^ (Scheme 3, 8 to 11) and (ii) stereoselective reduction of a C1-ketone intermediate to yield primarily the 1β-hydroxy epimer under Luche reduction conditions at −78 °C (Scheme 4, 13 to 14). Furthermore, 1α-hydroxytestosterone was accessed in 3 steps from commercially available starting materials (Schemes 1 and 2, 6 to 9), which contrasts from the previously reported synthesis of 1α-hydroxytestosterone involving 9 steps.^8^ The C1-borylation strategy can be used to access other naturally occurring steroids,^19^ including those that are not commercially available and have important biological applications.^20,21^

## Conflicts of interest

There are no conflicts to declare.

## Supplementary Material

OB-023-D5OB01218J-s001

OB-023-D5OB01218J-s002

## Data Availability

The data that support the findings of this study are available on request from the corresponding author, F. K. Y. Supplementary information: to show the experimental details (procedures, NMR, mass spectrometry, IR) to synthesize the compounds in the main text (Section S1), optimization of the borylation of boldione (Section S2, 6 to 7), optimization of the stereoselective reduction of the C1-ketone to the 1β-hydroxy product (Section S3, 13 to 14), and X-ray structures of the synthesized compounds (Section S4, 7, 9, 10, 12, 13, and 15. See DOI: https://doi.org/10.1039/d5ob01218j. CCDC 2448105, 2448104, 2448099, 2448100, 2448103 and 2448102 contain the supplementary crystallographic data for this paper.^22*a*–*f*^
