# Supplementary material for: A synthesis of 1β-hydroxytestosterone, a metabolite of xenobiotic human cytochrome P450 enzymes, beginning with a borylation of boldione
Source: Org Biomol Chem. 2025 Sep 9;23(42):9618–23. doi: 10.1039/d5ob01218j (PMC12679349; doi:10.1039/d5ob01218j)
Supplement: OB-023-D5OB01218J-s001 [file OB-023-D5OB01218J-s001.pdf]

Supplementary Information (SI) for Organic & Biomolecular Chemistry.
This journal is © The Royal Society of Chemistry 2025

Supplementary Information File

A Synthesis of 1β-Hydroxytestosterone, a Metabolite of Xenobiotic Human Cytochrome P450 Enzymes, Beginning with a Borylation of Boldione

Anna I. Elizondo,[1] Kevin D. McCarty,[2] Hadi D. Arman,[1] F. Peter Guengerich,[2] Francis K. Yoshimoto[1]

1: Department of Chemistry, the University of Texas at San Antonio (UTSA), San Antonio, Texas 78249, United States

2: Department of Biochemistry, Vanderbilt University School of Medicine, Nashville, Tennessee 37232-0146, United States

Anna I. Elizondo	https://orcid.org/0009-0002-4949-1288
Kevin D. McCarty	https://orcid.org/0000-0003-0027-9120
Hadi D. Arman	https://orcid.org/0000-0002-9084-560X
F. Peter Guengerich	https://orcid.org/0000-0002-7458-3048
Francis K. Yoshimoto	https://orcid.org/0000-0002-2308-2999

Table of Contents – Supplementary Information File

1. Synthesis of Compounds in the Main Text (Synthesis of 1β-Hydroxytestosterone, **2**) S3

1.1. Step 1 (**6** to **7**): Borylation of Boldione S7

1.2. Step 2 (**7** to **8**): Oxidation of C1-Borylated Steroid S11

1.3. Step 3 (**8** to **11**): Reduction of 3,17-Diketone S15

1.4. Step 4 (**11** to **12**): C3-Protection of 1,3,17-Triol with TBSCl S19

1.5. Step 5 (**12** to **13**): Oxidation of 1,17-Diol S23

1.6. Step 6 (**13** to **14**): Stereoselective Reduction of 1,17-Diketone S27

1.7. Step 7 (**14** to **15**): Deprotection of C3-TBS with TBAF S31

1.8. Step 8 (**15** to **2**): Regioselective Oxidation of Triol **15** S35

1.9. Synthesis of 1α-Hydroxytestosterone (Compound **9**) S39

2. Optimization of Step 1: 1,4-Borylation (Table 1 from Main Text) S43

3. Optimization of Step 6: Stereoselective Reduction of C1-ketone (Table 2 from Main Text) S46

4. X-Ray Structures of Synthesized Compounds S50

5. References for the SI file S54

1. Synthesis of Compounds in the Main Text (Synthesis of 1β-Hydroxytestosterone, 2)

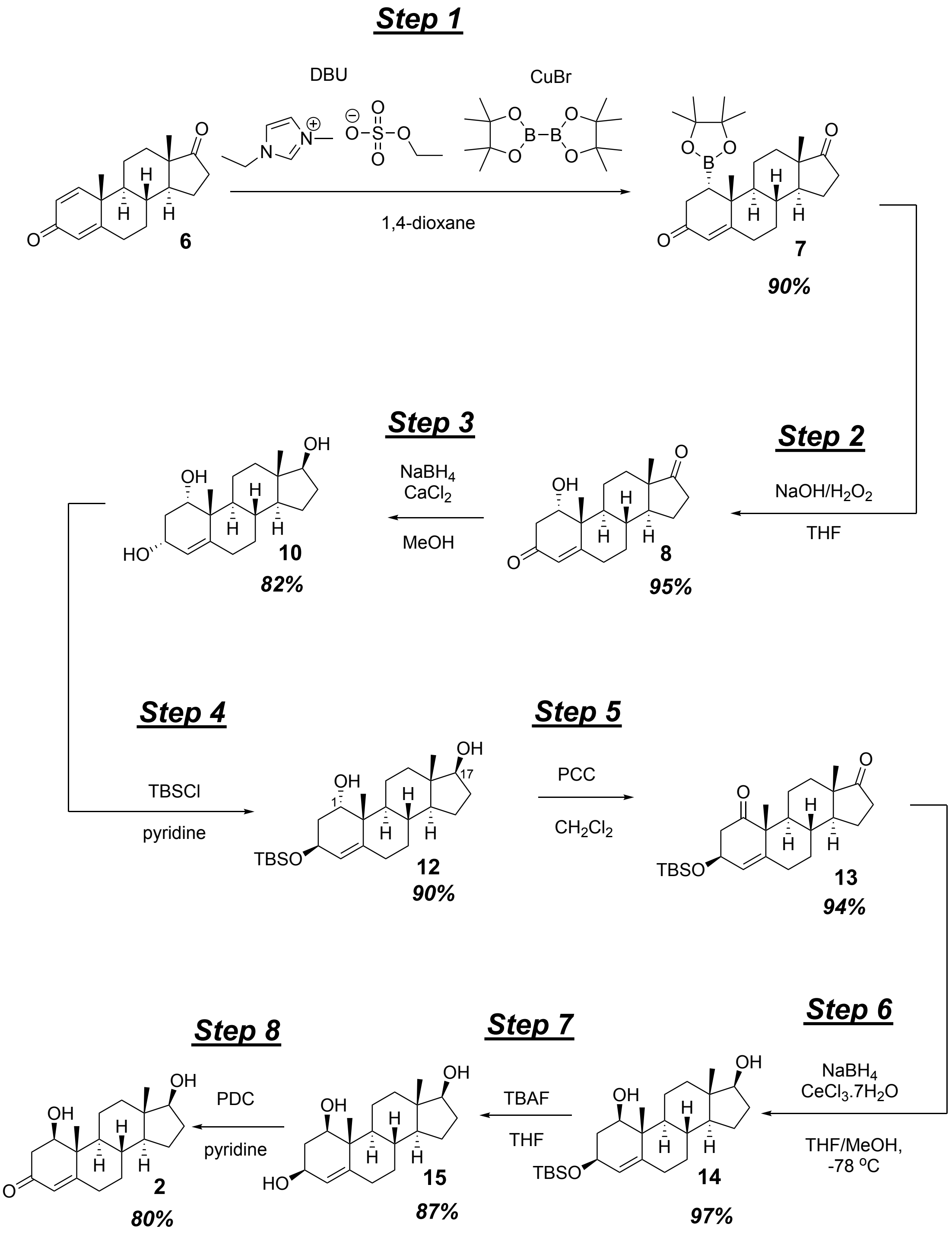

Figure S1-1. The 8-step synthesis of 1β-hydroxytestosterone (**2**) from boldione (**6**) presented in the main text.

1. Synthesis of Compounds in the Main Text (Synthesis of 1β-Hydroxytestosterone, 2)

NMR Spectroscopy: A Bruker (Billerica, MA) NMR spectrometer (500 MHz) was used to record NMR spectra of the synthesized intermediates. Deuterochloroform ($CDCl_3$, Cambridge Isotope Laboratories, Tewksbury, MA), dimethyl sulfoxide-d_6 (d_6-DMSO, Cambridge Isotope Laboratories, Tewksbury, MA) and deuterium oxide (D_2O, Aldrich Chemistry, St. Louis, MO) were used as the solvent for NMR spectra. Deuterochloroform was referenced to δ 7.26 ppm and δ 77.16 ppm for the ^{1}H NMR and ^{13}C NMR spectra. Dimethyl sulfoxide-d_6 was referenced to δ 2.50 ppm and δ 39.52 ppm for the ^{1}H NMR and ^{13}C NMR spectra, respectively.[1]

IR Spectroscopy: The Infrared (IR) spectroscopy of each compound was measured on a QATR-S IRSpirit Instrument (Shimadzu, Kyoto, Japan) using LabSolutions IR software Version 2.25 (Shimadzu Corporation, Kyoto, Japan).

Solvents for reactions were obtained from Fisher Scientific (Hampton, NH).

TLC: Thin-layer chromatography (TLC) plates (silica gel, Supelco, Darmstadt, Denmark) and a ceric ammonium molybdate stain (235 ml of H_2O, 15 ml of H_2SO_4, 12 g of ammonium molybdate, 0.5 g of ceric ammonium molybdate) was used to visualize compounds on TLC plates. Silica gel (40–63 µm, 60 Å) was purchased from SiliCycle (Quebec, Canada).

1. H.E. Gottlieb, V. Kotlyar, A. Nudelman, *J. Org. Chem.*, 1997, 62, 7512-7515.

1. Synthesis of Compounds in the Main Text (Synthesis of 1β-Hydroxytestosterone, 2)

UPLC-HRMS Analysis of Steroids: Steroid solid (roughly a few mg) was dissolved in CH_3OH (2 ml) and diluted 20-fold, giving a concentration of roughly ~500 μM (~5 mg of a 300-400 Da molecule dissolved in 2 mL is roughly 5-10 mM). Solutions (10 μL) were injected using a Waters Acquity UPLC on a 2.1 mm × 100 mm (1.7 μm) Acquity BEH octadecylsilane (C_{18}) column (part number: 186002352) equipped with a VanGuard BEH (C_{18}, 1.7 μm) guard column (part number: 186003975) with the following liquid chromatography conditions: the mobile phase composition was 0.1% HCO_2H in H_2O (solution A) and 0.1% HCO_2H in CH_3CN (solution B). The gradient separation method was as follows (expressed as %B, v/v): 0 min, 50%; 0.5 min, 50%; 3 min, 100%; 4.5 min, 100%; 4.6 min, 50%; 6 min, 50% (flow rate 0.3 mL min^{-1}). Column eluate was subjected to either heated electrospray ionization (HESI) ionization or atmospheric pressure chemical ionization (APCI) using a Thermo Fisher Scientific LTQ XL Orbitrap mass spectrometer operating in the positive-ion mode in the Vanderbilt Mass Spectrometry Research Core Facility. HESI data were collected with a resolution setting of 60,000 and scanning from *m/z* 100-500 using the following conditions: vaporizer temperature (ºC) 300, sheath gas flow rate (arb) 40, aux gas flow rate (arb) 10, sweep gas flow rate (arb) 0.01, spray voltage (kV) 5, capillary temperature (ºC) 275, and capillary voltage (V) 35. APCI-HRMS data were collected with the following parameter modifications: vaporizer temperature (ºC) 350, aux gas flow rate (arb) 5, and capillary voltage (V) 50. The mass spectrometer was calibrated prior to data collection with Pierce LTQ ESI Positive Ion Calibration Solution, part number: 88322, Thermo Scientific). Data were processed using Xcalibur QualBrowser (Thermo Fisher Scientific) software (version 2.0.7).

1. Synthesis of Compounds in the Main Text (Synthesis of 1β-Hydroxytestosterone, 2)

*A consistent signal at *m/z* 312 is observed in some of the LCMS runs that have less signal to noise. This signal is attributed to the mass of polypropylene glycol (PPG, ubiquitous polyether, *m/z* 289) adducted to sodium.

1.1. Step 1 (6 to 7): Borylation of Boldione

Experimental Procedure for 1α-Pinacolatoboryl-androst-4-en-3,17-dione (Compound **7**)

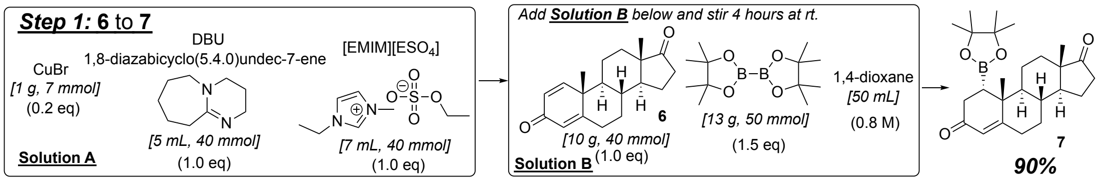

CuBr (1.01 g, 7.03 mmol, 0.2 eq), DBU (5.25 mL, 35.16 mmol, 1.0 eq), and 1-ethyl-3-methylimidazolium ethyl sulfate (6.7 mL, 35.16 mmol, 1.0 eq) were added and left to stir at room temperature for two minutes. Boldione (**6**) (10.0 g, 35.16 mmol, 1.0 eq) and Bis(pinacolato)diboron (13.4 g, 52.74 mmol, 1.5 eq) were added as a solution in 1,4-dioxane (50 mL). The reaction was stirred at rt for four hours and monitored by TLC. The reaction was then diluted with 100 mL of EtOAc and transferred to a separatory funnel and washed with H_2O (3 x 100 mL). The organic layer was then concentrated under reduced pressure to yield a green oil. The crude material was dissolved with DCM and purified through silica gel column chromatography (gradient from 100% hexanes to 100% ethyl acetate) to afford 1α-pinacolatoboryl-androst-4-en-3,17-dione (**7**) as a white solid (12.976 g, 31.5 mmol, 90%). mp of **7**: 162-188 °C. R_f = 0.610 (ethyl acetate:hexanes, 1:1, v/v). $[\alpha]^{20}_D$ – 0.043 [0.0025 g/10 mL in $(CH_3)_2CO$]; IR (neat) 2931.5, 1735.6, 1667.6, 1390.0, 1379.3, 1371.8, 1328.2, 1264.9, 1248.5, 1209.5, 1201.6, 1140.91, 975.66, 858.9, 731.8, 702.4, 676.1, 669.5 cm^{-1}; ^{1}H NMR (500 MHz, $CDCl_3$) 5.71 (s, 1H, H-4), 2.61 (m, 1H, H-2), 2.45 (m, 1H, H-16), 2.35 (m, 2H, H-6), 2.2 (m, 1H, H-2), 2.08 (m, 1H, H-16), 1.97 (m, 1H, H-15), 1.87 (m, 2H, H-12), 1.77 (m, 1H, H-11), 1.67 (m, 2H, H-1), 1.55 (m, 1H, H-15), 1.43 (m, 1H, H-11), 1.26 (s, 3H, H-19), 1.22 (m, 1H, H-14), 1.21 (m, 1H, H-9), 1.19 (m ,1H, H-7), 1.13 (s, 12H, H-C_B), 0.89 (s, 3H, H-18); ^{13}C NMR (125 MHz, $CDCl_3$) 220.72, 198.77, 168.03, 124.87, 83.42, 51.88, 51.31, 47.62, 40.36, 35.88, 35.79, 35.47, 33.99, 32.69, 32.00, 31.37, 30.84 (broad signal due to ^{13}C-boron coupling), 30.28, 30.11, 29.77, 29.44, 24.83, 24.69, 21.82, 20.48, 19.67, 13.85. HRMS of **7** (HESI) calculated for $[C_{25}H_{38}BO_4]^+$: 413.2858, $[MH]^+$; found: 413.2860. 1α-Pinacolatoboryl-androst-4-en-3,17-dione (compound **7**) was crystallized through slow evaporation of 1:1 ethyl acetate/hexanes (v/v). CCDC deposit number of **7**: 2448105.

1.1. Step 1 (6 to 7): Borylation of Boldione

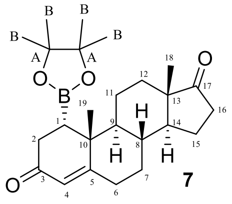

The 2 carbons labeled A are diastereotopic.

1α-Pinacolatoboryl-androst-4-en-3,17-dione (compound **7**)

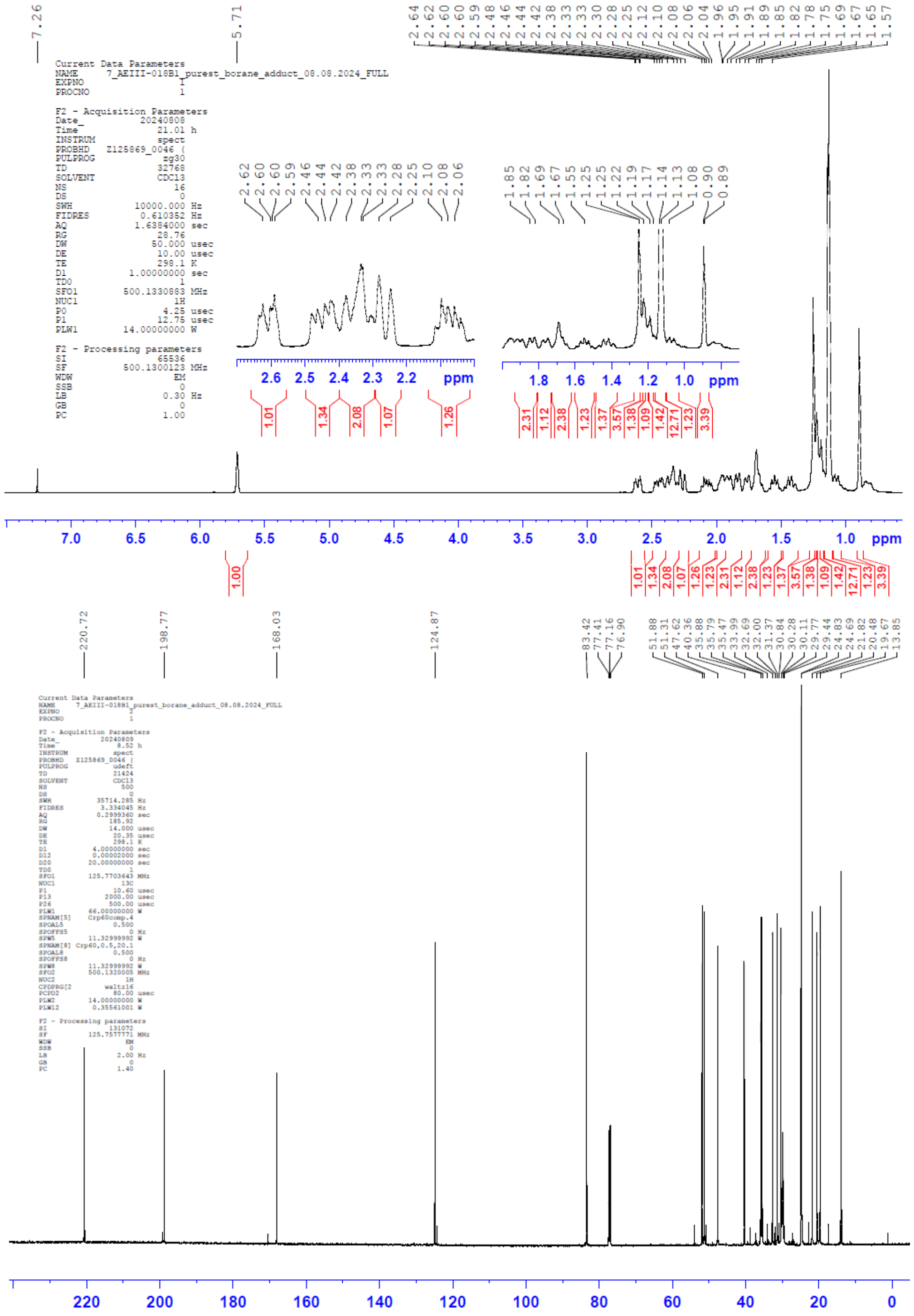

Figure S1.1-1. ^{1}H and ^{13}C NMR spectra (top and bottom) of compound **7**. https://nmrxiv.org/S856

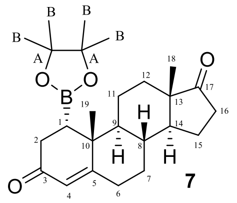

7

The 2 carbons labeled A are diastereotopic.

1.1. Step 1 (6 to 7): Borylation of Boldione

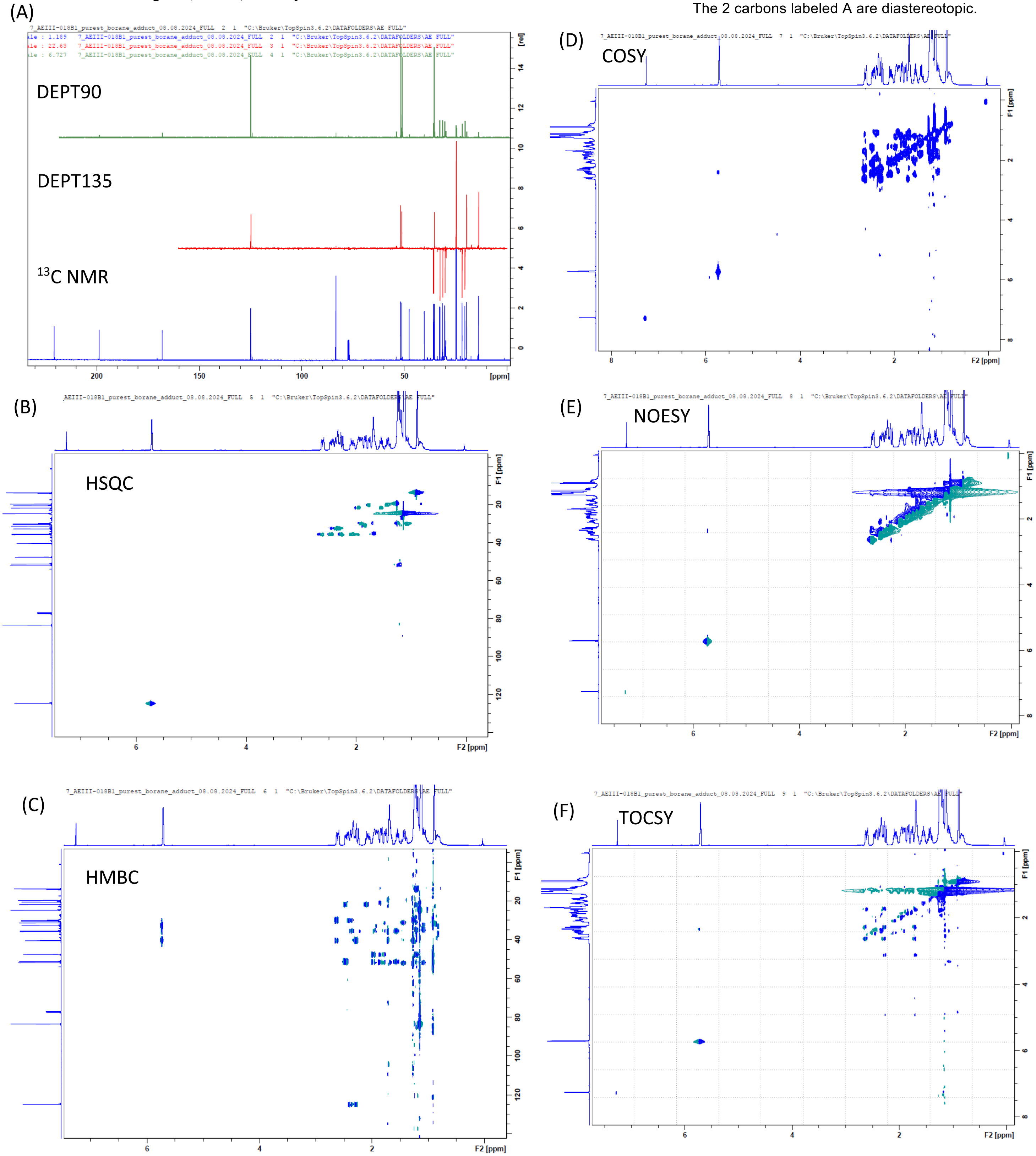

Figure S1.1-2: (A) DEPT90, DEPT135, ^{13}C NMR, (B) HSQC, (C) HMBC, (D) COSY, (E) NOESY, (F) TOCSY of 7.

1.1. Step 1 (6 to 7): Borylation of Boldione

Table S1.1-1. NMR assignment of 1α-Pinacolatoboryl-androst-4-en-3,17-dione (Compound **7**)

https://nmrxiv.org/S856

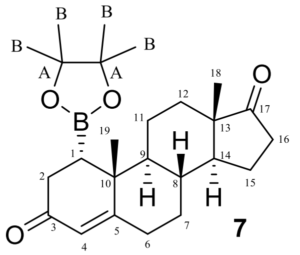

7

The 2 carbons labeled A are diastereotopic.

Position	^{13}C	^{1}H	Interactions
1 (-CH-)	30.6 (broad)	1.71	^{13}C signal is based on HSQC (^{13}C, J = 83 Hz)
2 (-CH2-)	35.7	2.64, 2.26	1.71-COSY-2.64
3 (C)	198.6		2.64+2.29-HMBC-198.6,
4 (CH)	124.8	5.71	5.71-HMBC-168.4
			124-HMBC-2.38 (C6), 2.28 (C2)
5 (C)	168.4		168-HMBC-2.39 (C6), 1.73 (C1), 1.27 (C9)
6(-CH2-)	32.7	2.38	2.38-COSY-1.09, 2.38-HMBC-35.5(C8 close to C16)
7 (-CH2-)	30.0	1.09, 1.91	
8(-CH-)	35.5	1.70	35.5-HMBC-2.38(C6)
9 (-CH-)	51.9	1.27	
10 (C)	40.1		40.1-HMBC-1.23
11 (-CH2-)	20.4	1.77, 1.43	Based on ruling out the signals
12 (-CH2-)	30.8	1.84	
13(C)	47.7**		47.7-HMBC-1.98
14(-CH-)	51.8**	1.23	
15(-CH2-)	21.2	1.97, 1.56	Based on 47.7-HMBC-1.98 AND 1.56-COSY-2.49
16(-CH2-)	35.6	2.1, 2.48	220-HMBC-2.1,2.48
17(C)	221.1		220-HMBC-2.1,2.48
18(-CH3-)	13.7	0.92	singlet, 3H, 0.92-HMBC-30.8, 47.7** (C), 51.8** (CH)
19(-CH3-)	19.6	1.23	19.6-HMBC-1.72 , 1.27
			1.23-HMBC-51.9 (-CH-), 40.1 (C), 30.0

1.2. Step 2 (7 to 8): Oxidation of C1-Borylated Steroid

Experimental Procedure for (Compound **8**)

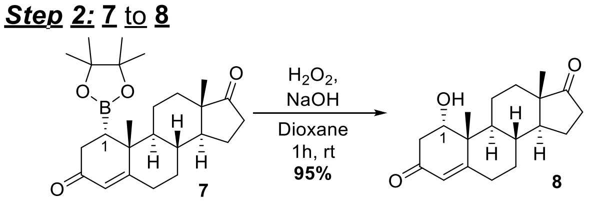

1α-Pinacolatoboryl-androst-4-en-3,17-dione (Compound **7**, 3.0 g, 7.28 mmol, 1.0 eq) was dissolved with 1,4-dioxane (40 mL) and H_2O_2 (0.83 mL, 7.28 mmol, 1.0 eq) and NaOH (291 mg, 7.28 mmol, 1.0 mol eq) dissolved in water (2.0 mL) were added. The reaction was left stirring at RT for one hour and monitored by TLC. The reaction mixture was then washed with H_2O (100 mL) and extracted with EtOAc (3 x 100 mL). The organic layer was concentrated under reduced pressure to yield a clear oil. The crude was purified through silica gel column chromatography (100% hexanes in ethyl acetate to 100% ethyl acetate) to afford 1α-hydroxy-androst-4-en-3,17-dione (Compound **8**) as a white solid (2.081 g, 6.91 mmol, 95%). mp of **8**: 166-168 °C. Literature value[2] for the mp of **8**: 186-188 °C. R_f = 0.146 (ethyl acetate:hexanes, 1:1, v/v). $[\alpha]^{20}_D$ = 0.0029 [0.029 g/10 mL in $(CH_3)_2CO$]; IR (neat) 3051.4, 2933.0, 2857.4, 2156.9, 1734.7, 1670.1, 1663.4, 1265.4, 1054.2, 736.1, 703.3 cm^{-1}; ^1H NMR (500 MHz, $CDCl_3$): 5.82 (s, 1H, H-4), 4.12 (t, J=3.01 Hz, 1H, H-1), 2.76 (dd, J=17.92, 2.51 Hz, 1H, H-2), 2.58 (dd, J= 17.21, 2.87 Hz, 1H, H-2), 2.49 (m, 2H, C16, H-6), 2.41 (m, 1H, H-6), 2.13 (m, 1H, H-16), 2.01 (m, 1H, H-15), 1.94 (m, 1H, H-7), 1.86 (m, 1H, H-12), 1.77 (m, 2H, H-8, H-9), 1.72 (m, 1H, H-11), 1.59 (m, 1H, H-15), 1.47 (m, 1H, H-11), 1.39 (m, 1H, H-14), 1.33 (m, 1H, H-12), 1.23 (s, 3H, H-19), 1.16 (m, 1H, H-7), 0.93 (s, 3H, H-18); ^{13}C NMR (125 MHz, $CDCl_3$) 220.66, 197.03, 166.74, 123.52, 71.80, 50.83, 47.54, 44.92, 43.24, 42.80, 35.77, 34.83, 32.75, 31.13, 29.85, 21.82, 19.66, 18.54, 13.70. HRMS of **8** (HESI) calculated for $[C_{19}H_{27}O_3]^+$: 303.1955, $[MH]^+$; found: 303.1956.

2. B. Pelc, J. Hodkova, *Collect. Czech. Chem. Commun.*, 1967, 32, 410-418.

1.2. Step 2 (7 to 8): Oxidation of C1-Borylated Steroid

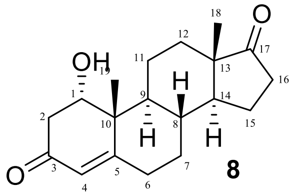

1α-Hydroxy-androst-4-en-3,17-dione (Compound **8**) https://nmrxiv.org/S869

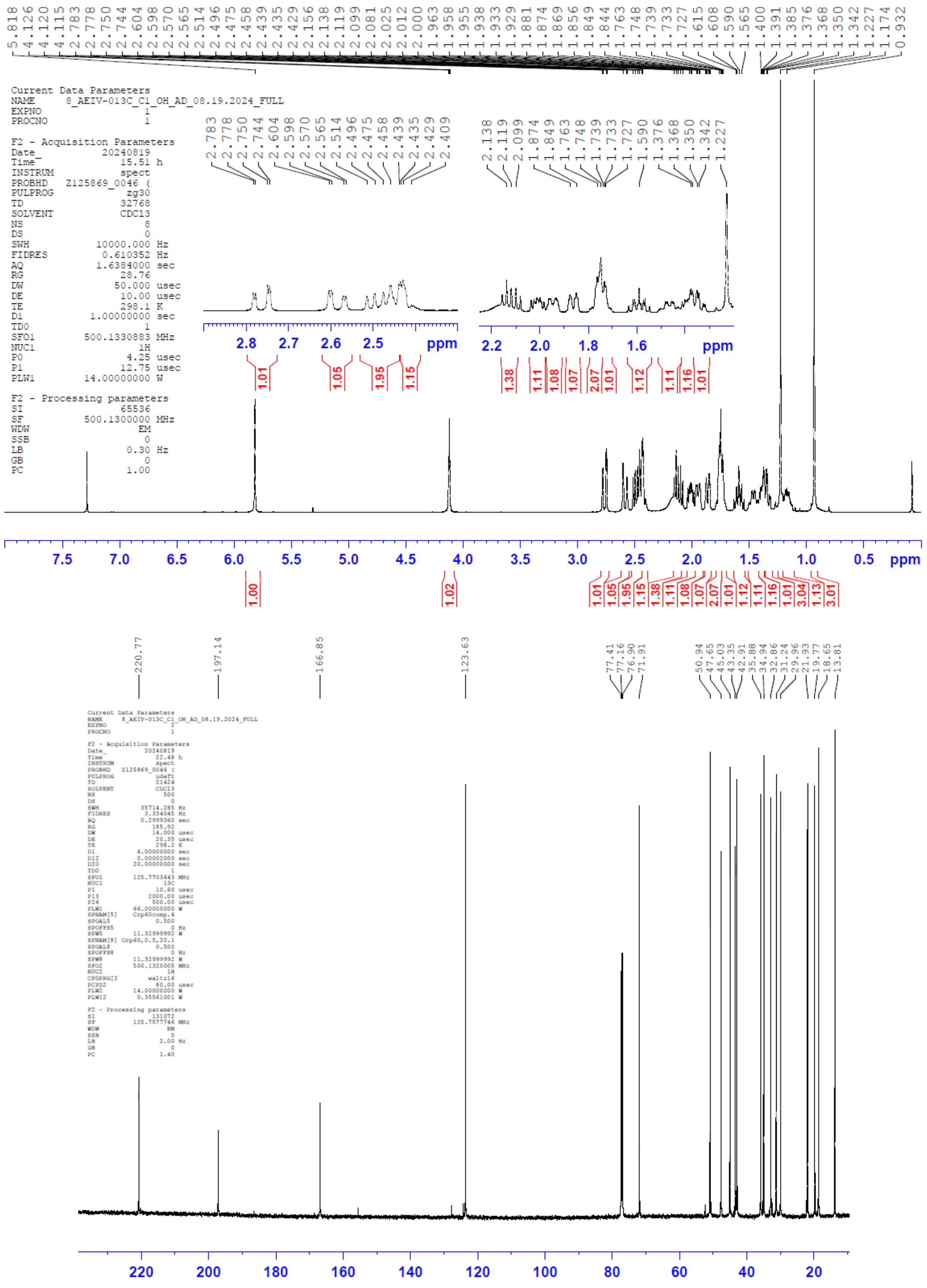

Figure S1.2-1. ^{1}H and ^{13}C NMR spectra (top and bottom) of compound **8**.

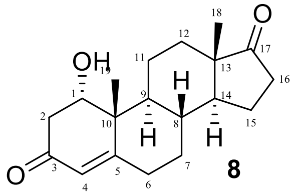

8

1.2. Step 2 (7 to 8): Oxidation of C1-Borylated Steroid

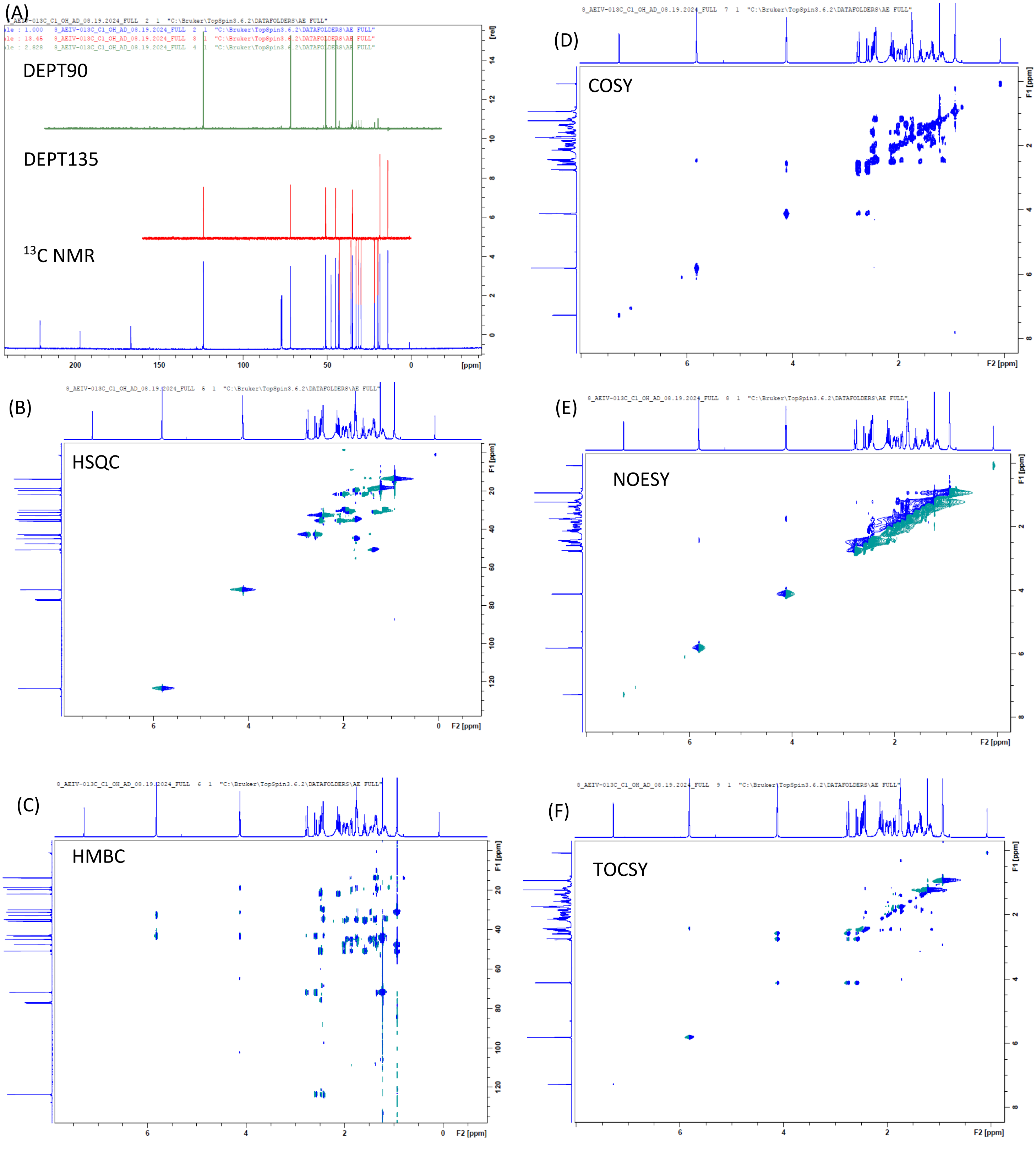

Figure S1.2-2: (A) DEPT90, DEPT135, ^{13}C NMR, (B) HSQC, (C) HMBC, (D) COSY, (E) NOESY, (F) TOCSY of 8.

1.2. Step 2 (7 to 8): Oxidation of C1-Borylated Steroid

Table S1.2-1. NMR assignment of 1α-Hydroxy-androst-4-en-3,17-dione (Compound **8**)

https://nmrxiv.org/S869

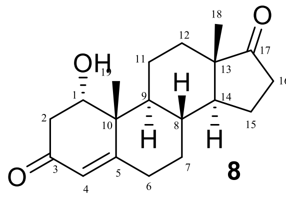

8

Position	^{13}C	^{1}H	Interactions
1(-CH-)	71.8	4.116	
2(-CH2-)	42.8	2.76, 2.56	
3	197.18		197.8-HMBC-4.13, 2.78, 2.57
4(-CH=)	123.64	5.82	5.82-HMBC-43.1(C2), 33.1, 19.7
5	166.9		166.9-HMBC-4.12(C1), 2.44(C6), 1.22(C19)
6(-CH2-)	32.6	2.44, 2.46	32.8-HMBC-5.82
			2.42-COSY-1.17
			2.43-HMBC-34.7(C8)
7(-CH2-)	29.7	1.93, 1.15	
8(-CH-)	34.7	1.74	34.7-HMBC-2.43(C6)
9(-CH-)	44.95	1.75	
10	43.8		43.8-HMBC-1.23
11(-CH2-)	19.7	1.73, 1.45	Through elimination
12(-CH2-)	30.7	1.85, 1.33	
13	47.6		
14(-CH-)	51.3	1.38	
15(-CH2-)	21.8	2.00, 1.58	
16(-CH2-)	35.7	2.45, 2.09	
17	219		219-HMBC-2.48(C16), 2.14(C16), 2.00
18(-CH3)	13.9	0.94	0.95-HMBC-219(C17)
			0.95-HMBC-31.6(C12, DEPT135), 47.6, 51.3(C14)
19(-CH3)	18.4	1.23	1.23-HMBC-44.2(C10), 71.9(C1), 167.5(C5)

1.3. Step 3 (8 to 11): Reduction of 3,17-Diketone

Experimental Procedure for Androst-4-en-1α,3β-17β-triol (Compound 11)

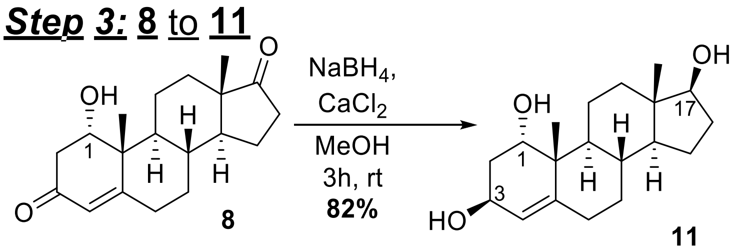

A solution of 1α-hydroxy-androst-4-en-3,17-dione (Compound **8**, 1.709 g, 5.65 mmol, 1.0 eq) and $CaCl_2$ (12.381 g, 56.5 mmol, 10.0 eq) in MeOH (60 mL) was stirred at room temperature for 30 minutes. $NaBH_4$ (2.1379 g, 56.5 mmol, 10.0 eq) was added in small portions, and the reaction was left stirring at room temperature for three hours. The reaction mixture was washed with H_2O (100 mL) and extracted with EtOAc (5 x 100 mL). The organic layer was concentrated under reduced pressure to yield a clear oil. The crude was purified through silica gel column chromatography (1:1 ethyl acetate in hexanes to 1:1 ethyl acetate in MeOH) to afford androst-4-en-1α,3β-17β-triol (**Compound 11**) as a white solid (1.422 g, 4.66 mmol, 82%). mp of **11**: 223-233 °C. Literature value of the mp for **11**: 236-237 °C.[3] R_f= 0.2 (100% ethyl acetate, v/v). $[\alpha]^{20}_D$ = 0.006 [0.0036 g/10 mL in MeOH]; IR (neat) 2948.7, 29.27.3, 23.75, 1653.4, 1559.2, 1507.9, 1255.4, 1065.6, 835.9, 774.6, 669.1 cm^{-1}; ^1H NMR (500 MHz, $CDCl_3$) 5.39 (s, 1H, H-4), 4.48 (m, 1H, H-3), 3.88 (d, J = 3.79 Hz, 1H, H-1), 3.67 (t, J = 8.58 Hz, 1H, H-17), 2.29 (m, 1H, H-7), 2.23 (m, 1H, H-15), 2.14 (m, 1H, H-15), 2.06 (m, 1H, H-16), 1.85 (m, 1H, H-12), 1.74 (m, 1H, H-6), 1.70 (m, 1H, H-2), 1.64 (m, 1H, H-7), 1.52 (m, 1H, H-11), 1.49 (m, 1H, H-14), 1.44 (m, 1H, H-11), 1.40 (m, 1H, H-7), 1.37 (m, 1H, H-7), 1.30 (m, 1H, H-12), 1.21 (m, 1H, H-16), 1.11 (s, 3H, H-19), 1.03 (m, 1H, H-9), 0.95 (m, 1H, H-6), 0.80 (s, 3H, H-18); ^{13}C NMR (125 MHz, $CDCl_3$) 143.81, 122.65, 81.97, 72.68, 64.58, 50.86, 45.75, 43.03, 41.60, 36.57, 36.06, 35.88, 32.43, 31.75, 30.59, 23.59, 20.53, 20.15, 11.21. HRMS of **11** (HESI) calculated for $[C_{19}H_{27}O_3]^+$: 303.1955, $[MH]^+$; found: 303.1956. Treatment of ketone **8** with DIBAL-H resulted in the elimination of the C1-hydroxy group to yield boldione (**6**).

3. W.R. Benn, F. Colton, R. Pappo, *J. Am. Chem. Soc.*, 1957, 79, 3920.

1.3. Step 3 (8 to 11): Reduction of 3,17-Diketone

Androst-4-en-1α,3β-17β-triol (**Compound 11**)

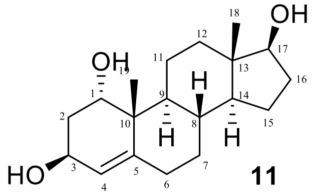

11

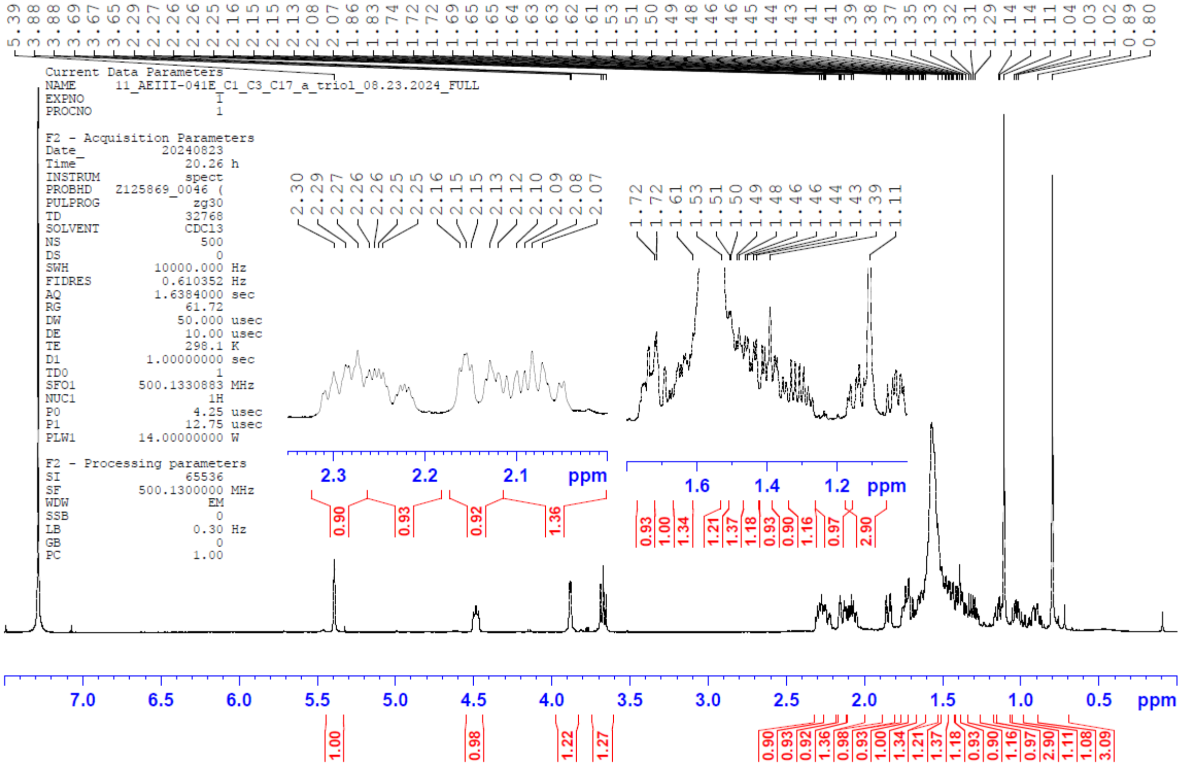

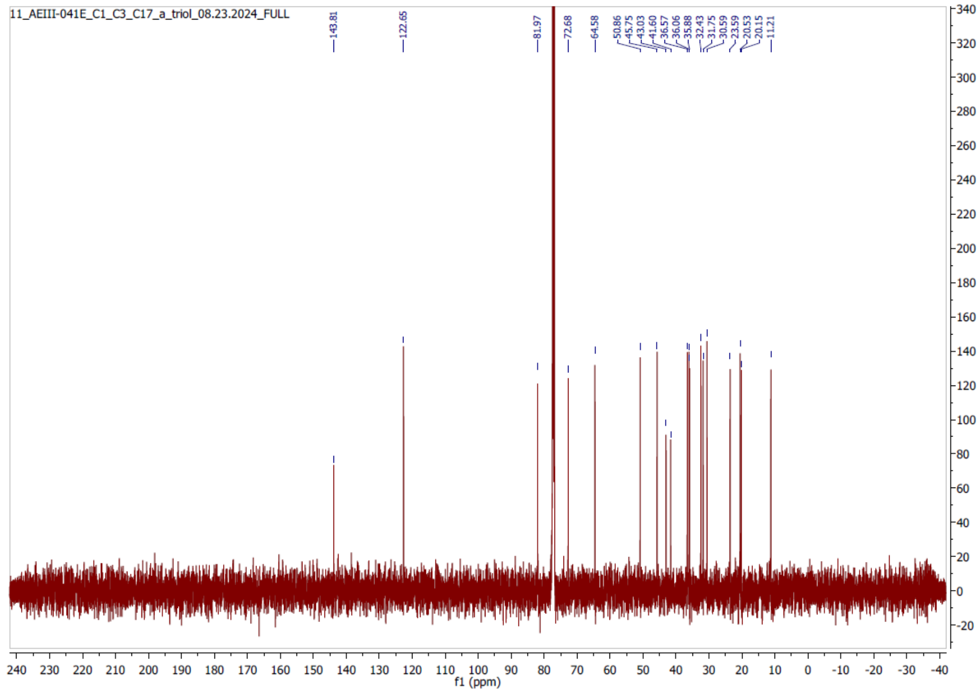

Figure S1.3-1. ^{1}H and ^{13}C NMR spectra (top and bottom) of compound **11**.

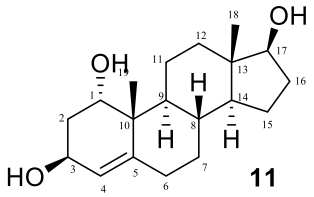

11

1.3. Step 3 (8 to 11): Reduction of 3,17-Diketone

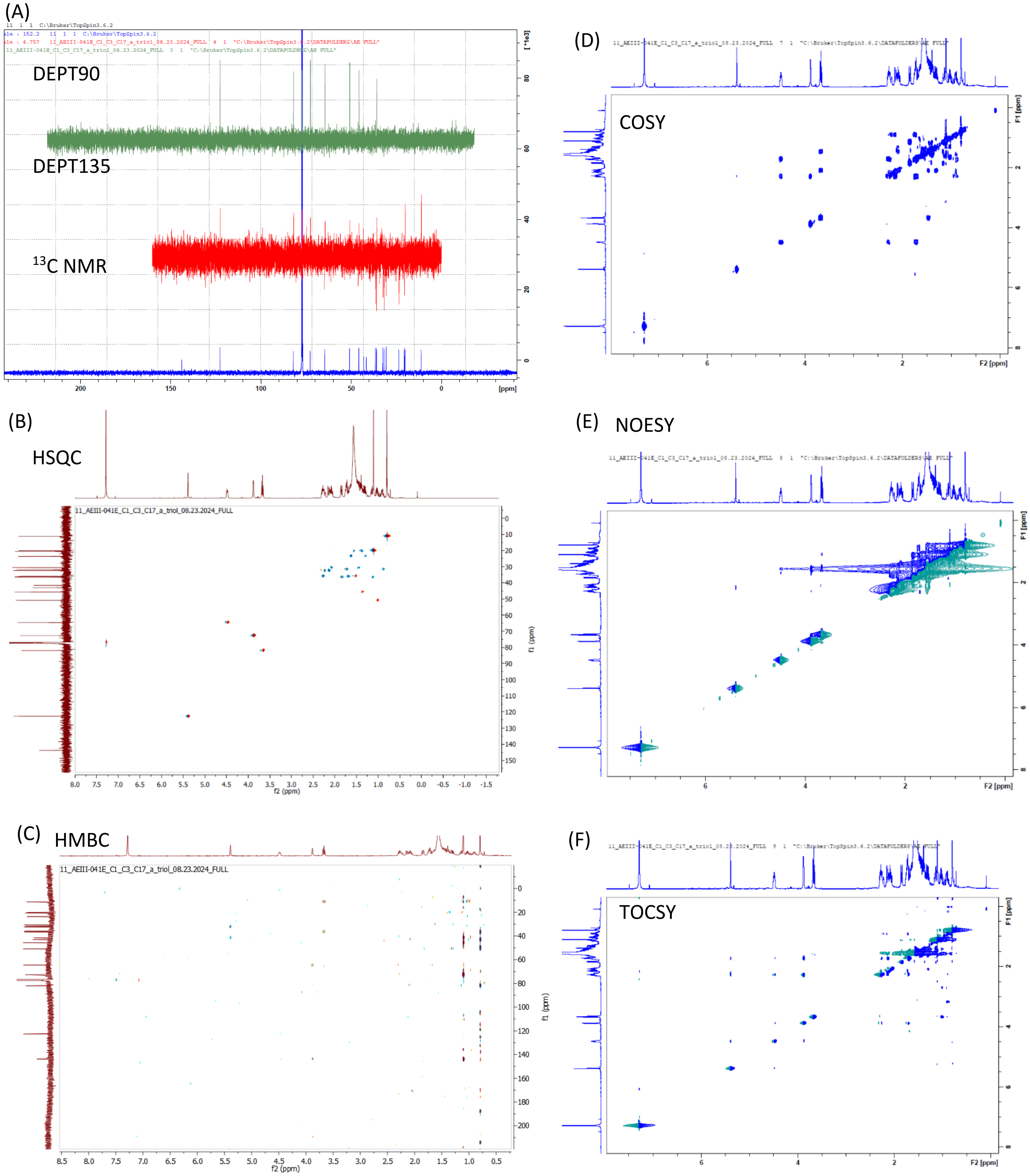

Figure S1.3-2: (A) DEPT90, DEPT135, ^{13}C NMR, (B) HSQC, (C) HMBC, (D) COSY, (E) NOESY, (F) TOCSY of 11.

1.3. Step 3 (8 to 11): Reduction of 3,17-Diketone

Table S1.3-1. NMR assignment of Androst-4-en-1α,3β-17β-triol (Compound **11**).

https://nmrxiv.org/S868

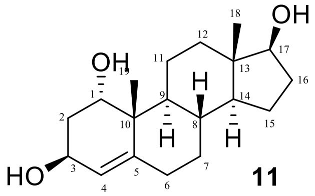

11

Position	^{13}C	^{1}H	Interactions
1(-CH-)	72.71	3.876	72.71-HMBC-1.098, (2.2878)
			3.876-HMBC-64.9, 23.6, 144.2, 36.1
2(-CH2-)	36.1	1.69, 1.72	
3(-CH-)	64.56	4.47	4.4792-HMBC-122.6, 144.1, 4.47-COSY-2.32,1.71
4(-CH=)	122.6	5.39	5.41-HMBC-33.3,42.1 (6 or 2)
5	143.7		41.51-HMBC-5.395, 1.114, 0.993
6(-CH2-)	31.85	0.993, 1.74	31.85-HMBC-1.07
7(-CH2-)	23.46	1.62, 1.31	1.31-COSY-1.09
8(-CH-)	20.02	1.09	
9(-CH-)	51.1	1.0	RULE OUT
10	43.1		43.1-HMBC-1.12, 0.80
11(-CH2-)	19.8	1.42, 1.55	
12(-CH2-)	36.6	1.82, 1.12	1.82-COSY-1.11, 1.42
13	41.6		43.1-HMBC-1.12, 0.80
14(-CH-)	35.75	1.50	
15(-CH2-)	32.43	2.24, 2.14	
16(-CH2-)	30.7	2.07, 1.146	1.14-COSY-1.84
17(-CH-)	82.4	3.66	82.4-HMBC-1.146, 0.8
18(-CH3)	10.8	0.80	0.80-HMBC-36.6,43.0,50.6,82.4
19(-CH3)	20.3	1.11	1.11-HMBC-41.5, 45.7, 72.9, 144.0

1.4. Step 4 (11 to 12): C3-Protection of 1,3,17-Triol with TBSCl

Experimental Procedure for 3β-(*tert*-Butyldimethylsiloxy)-androst-4-en-1α,17β-diol (compound 12)

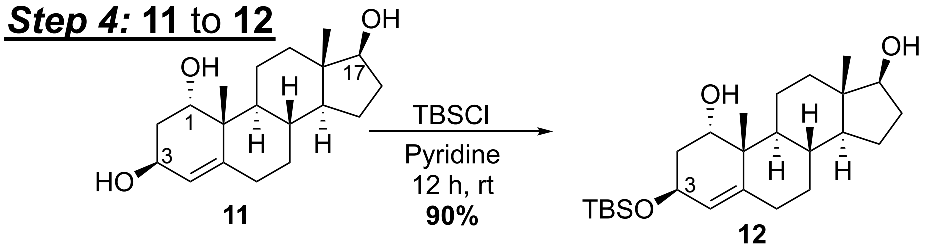

Androst-4-en-1α,3β-17β-triol (Compound **11**, 1.422 g, 4.657 mmol, 1.0 eq) was dissolved with molecular sieves dried pyridine (20 mL) and TBSCl (2.106 g, 13.97 mmol, 3.0 eq) was added. The reaction was left stirring at RT overnight. Reaction was washed with H_2O (100 mL) and extracted with ethyl acetate (3 x 100 mL) to yield a yellow tinted clear oil. The crude was purified through silica gel column chromatography (100% hexanes to 1:1 ethyl acetate in MeOH) to afford 3β-(*tert*-butyldimethylsiloxy)-androst-4-en-1α,17β-diol (compound **12**) as a yellow tinted solid (1.763 g, 4.1913 mmol, 90%). mp: 149-154 °C. R_f = 0.846 (100% ethyl acetate, v/v). $[\alpha]^{20}_D$ = 0.015 [0.0040 g/10 mL in $(CH_3)_2CO$]; IR (neat) 2951.5, 2941.6, 2930.1, 2373.8, 2322.43, 1684.8, 1653.4, 1559.2, 1507.9, 1259.7, 1058.5, 834.5, 764.6, 748.6, 669.1 cm^{-1} ; ^1H NMR (500 MHz, $CDCl_3$) 5.31 (s, 1H, H-4), 4.51 (t, J=10.12, 7.99 Hz, 1H, H-3), 3.84 (d, J=3.83 Hz, 1H, C1), 3.66 (t, J = 9.02, 9.72 Hz, 1H, H-17), 2.24 (m, 1H, H-6), 2.14 (m, 1H, H-9), 2.11 (m, 1H, H-2), 2.07 (m, 1H, H-6), 1.84 (m, 1H, H-15), 1.78 (m, 1H, H-16), 1.73 (m, 1H, H-2), 1.66 (m, 1H, H-12), 1.61 (m, 1H, H-7), 1.44 (m, 1H, H-7), 1.37 (m, 1H, H-11), 1.32 (m, 1H, H-11), 1.29 (m, 1H, H-12), 1.15 (m, 1H, H-15), 1.10 (s, 3H, H-19), 1.02 (m, 1H, H-14), 0.93 (s, 9H, Si-$C(CH_3)_3$), 0.90 (m, 1H, H-8), 0.85 (m, 1H, H-7), 0.80 (s, 3H, H-18), 0.12 (s, 3H), 0.11 (s, 3H); ^{13}C NMR (125 MHz, $CDCl_3$) 142.29, 123.60, 81.86, 72.81, 65.05, 50.77, 45.72, 42.90, 41.34, 36.47, 36.14, 35.76, 32.35, 31.69, 30.46, 29.71, 26.03, 23.46, 20.39, 19.81, 18.37, 11.07, 1.03, -4.48, -4.53. HRMS of **12** (HESI). Calculated for $[C_{25}H_{45}O_3Si]^+$: 421.3132, $[MH]^+$; found: 421.3128. 3β-(*tert*-Butyldimethylsiloxy)-androst-4-en-1α,17β-diol (compound **12**) was crystallized through slow evaporation of 100% EtOAc. CCDC # of **12**: 2448100.

1.4. Step 4 (11 to 12): C3-Protection of 1,3,17-Triol with TBSCl

3β-(*tert*-Butyldimethylsiloxy)-androst-4-en-1α,17β-diol (compound **12**)

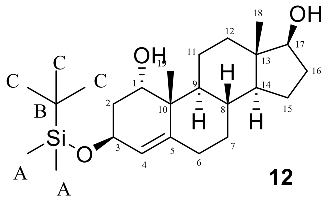

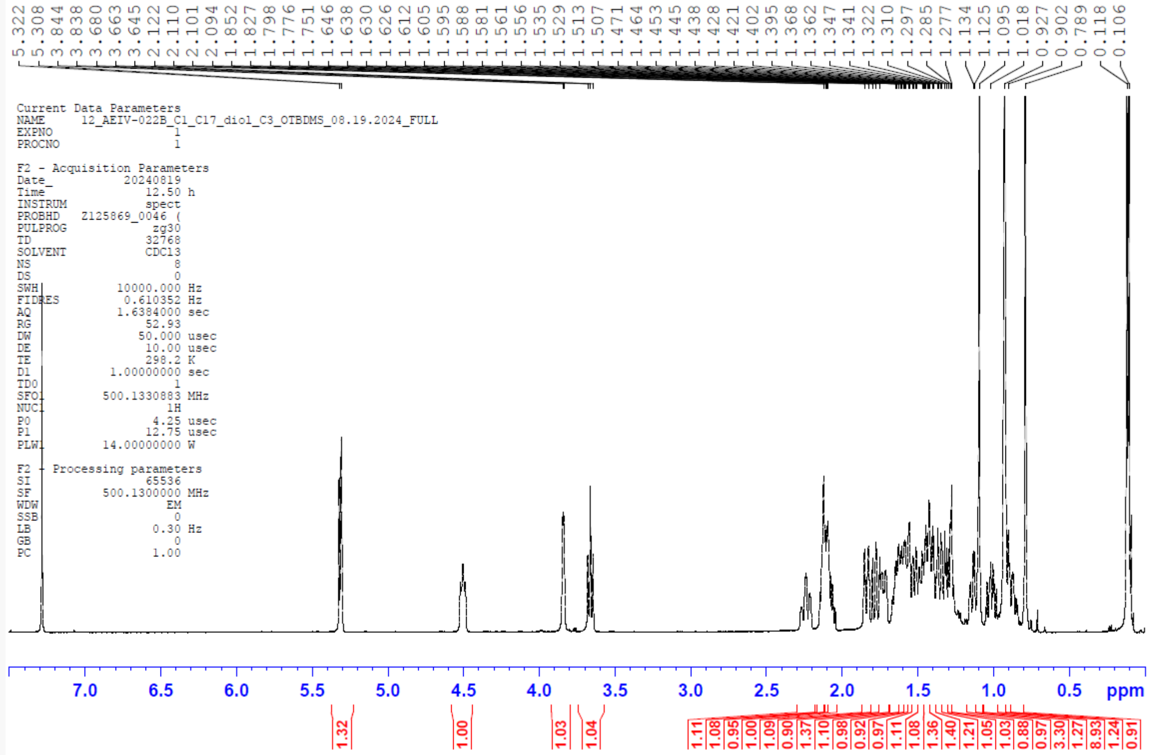

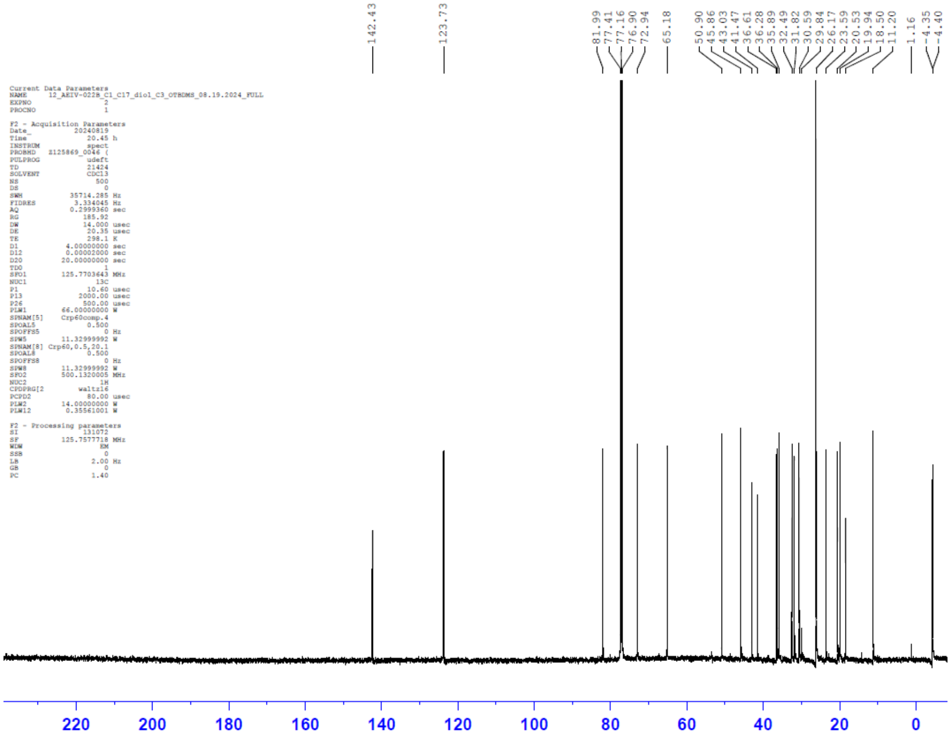

Figure S1.4-1. ^{1}H and ^{13}C NMR spectra (top and bottom) of compound **12**.

1.4. Step 4 (11 to 12): C3-Protection of 1,3,17-Triol with TBSCl

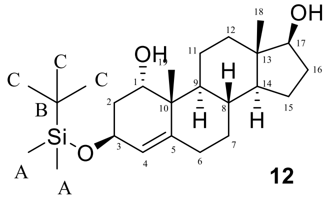

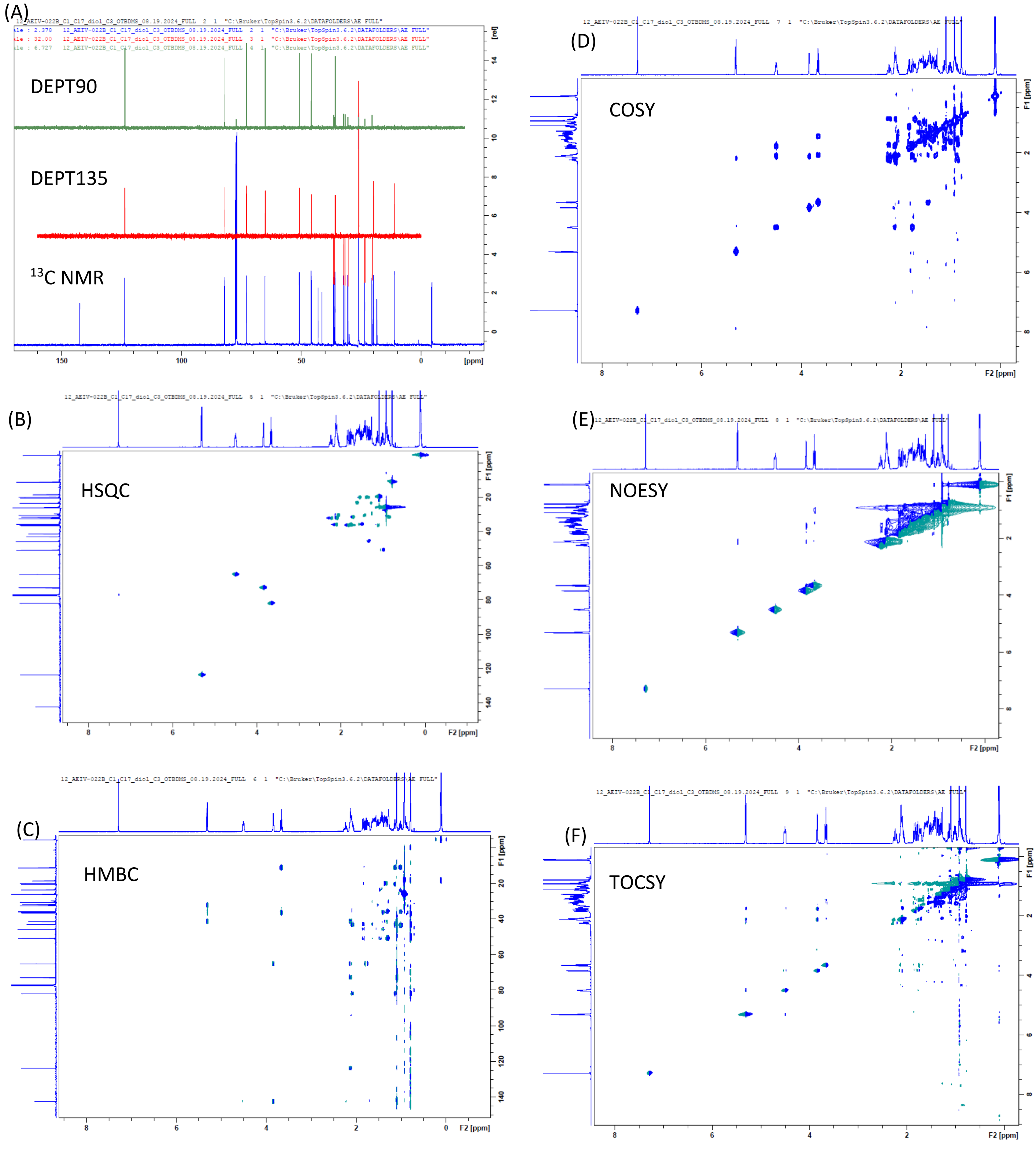

Figure S1.4-2: (A) DEPT90, DEPT135, ^{13}C NMR, (B) HSQC, (C) HMBC, (D) COSY, (E) NOESY, (F) TOCSY of 12.

1.4. Step 4 (11 to 12): C3-Protection of 1,3,17-Triol with TBSCl

Table S1.4-1. NMR assignment of 3β-(*tert*-Butyldimethylsiloxy)-androst-4-en-1α,17β-diol (Compound **12**)

https://nmrxiv.org/S867

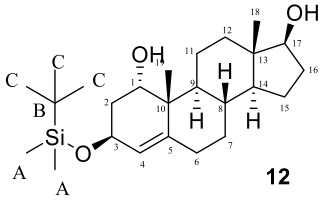

12

Position	^{13}C	^{1}H	Interactions
1(-CH-)	73.3	3.826	3.826-HMBC-65.3,142.6,
			3.826-COSY-2.12
2(-CH_2-)	36.22	2.12, 1.75	2.12-HMBC-124.3(4),73.1(1),65.1(3),41.6(10)
3(-CH-)	64.2	4.503	64.2-HMBC-3.854, 2.142, 2.101, 1.797,1.747
4(-CH=)	124.1	5.317	5.317-HMBC-32.44, 36.22, 41.53
5	142.5	-------	
6(-CH_2-)	32.44	2.214, 2.08	2.214-HMBC-124.0, 143.4, 32.4
7(-CH_2-)	30.45	0.84, 1.44	
8(-CH-)	19.9	1.02	Through elimination
9(-CH-)	35.89	2.15	
10	41.6	-------	41.6-HMBC-5.32, 2.12, 1.32, 1.35
11(-CH_2-)	20.4	1.32,1.35	
12(-CH_2-)	23.6	1.29, 1.63	23.6-HMBC-1.47,1.02,2.07
13	43.1	-------	
14(-CH-)	50.8	1.00	50.8-HMBC-0.79, 1.29(23.6), 1.32(46.1),
			1.00-HMBC-11.1(18), 23.6(12),35.89(8)
15(-CH_2-)	36.5	1.13, 1.82	(RULE OUT, DEPT135)
16(-CH_2-)	36.3	1.877, 1.09	1.877-HMBC-43.1(13), 50.8(14),
17(-CH-)	82.1	3.665	3.665-HMBC-11.3 (18), 36.3(16)
18(-CH_3)	11.2	0.789	0.789-HMBC-36.7(16), 43.1(13), 50.9(14), 82.0(17)
19(-CH_3)	19.89	1.095	1.09-HMBC-41.4(10),45.80(CH),73.0(1), 142.3(5)
A(Me)	26.0	0.934	
C(Si-C)	-4.39	0.16	

1.5. Step 5 (12 to 13): Oxidation of 1,17-Diol

Experimental Procedure for 3β-(*tert*-Butyldimethylsiloxy)-androst-4-en-1,17-dione (Compound 13)

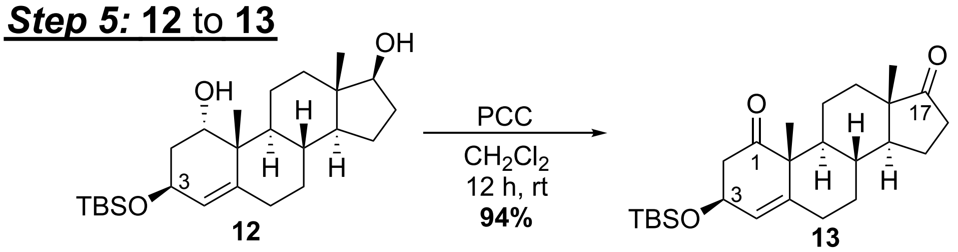

3β-(*tert*-Butyldimethylsiloxy)-androst-4-en-1α,17β-diol (Compound **12**) (1.763 g, 4.1913 mmol, 1.0 eq) was dissolved with molecular sieves dried DCM (20 mL). PCC (2.710 g, 12.57 mmol, 3.0 eq) was added to the solution and capped. The reaction was left stirring at RT overnight. Reaction was monitored through TLC. Reaction was directly loaded to a silica gel column chromatograph and purified (100% Hexanes to 80% Ethyl acetate in Hexanes) to afford 3β-(*tert*-butyldimethylsiloxy)-androst-4-en-1,17-dione (Compound **13**) as a white powdery solid (1.619 g, 3.95 mmol, 94%). mp: 125-133 °C. R_f=0.927 (ethyl acetate:hexanes, 1:1, v/v). $[\alpha]^{20}_D$ = -0.050 [0.0056 g/10 mL in $(CH_3)_2CO$]; IR (neat) 2927.29, 2855.96, 1738.97, 1706.16, 1362.36, 1244.38, 1054.22, 931.54, 827.40, 777.47, 666.20, 556.36 cm^{-1}; ^{1}H NMR (500 MHz, $CDCl_3$) 5.62 (d, J = 5.16 Hz, 1H, H-4), 4.44 (dd, J_1 = 8.80 Hz, J_2 = 4.52 Hz, 1H, H-3), 2.72 (dd, J_1 = 12.64 Hz, J_2 = 4.21 Hz, 1H, H-2), 2.49 (m, 1H, H-2), 2.44 (m, 1H, H-16), 2.34 (m, 1H, H-7), 2.19 (m, 1H, H-7), 2.06 (m, 1H, H-16), 1.94 (m, 2H, H-6), 1.45 (m, 1H, H-15), 1.40 (m, 1H, H-12), 1.30 (m, 1H, H-18), 1.25 (s, 3H, H-19), 1.22 (m, 1H, H-15), 1.18 (m, 1H, H-12), 1.05 (m, 1H, H-11), 0.88 (s, 3H, H-18), 0.84 (s, 9H, Si-$C(CH_3)_3$), 0.05 (s, 3H, Si-CH_3), 0.02 (s, 3H, Si-CH_3); ^{13}CNMR (125 MHz, $CDCl_3$) 220.53, 210.77, 147.94, 121.82, 67.20, 52.05, 51.32, 49.05, 47.92, 46.15, 35.84, 35.73, 32.09, 31.58, 31.29, 25.89, 25.79, 22.40, 21.83, 18.16, 15.80, 13.98, 1.16, -4.28, -4.63. HRMS of **13** calculated for $[C_{25}H_{39}O_3Si]^+$: 415.2663, $[MH-2H]^+$; found: 415.2655. 3β-(*tert*-Butyldimethylsiloxy)-androst-4-en-1,17-dione (Compound **13**) was crystallized through slow evaporation of 100% ethyl acetate. CCDC # of **13**: 2448100.

1.5. Step 5 (12 to 13): Oxidation of 1,17-Diol

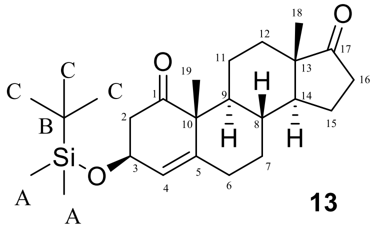

13

3β-(*tert*-Butyldimethylsiloxy)-androst-4-en-1,17-dione (Compound **13**)

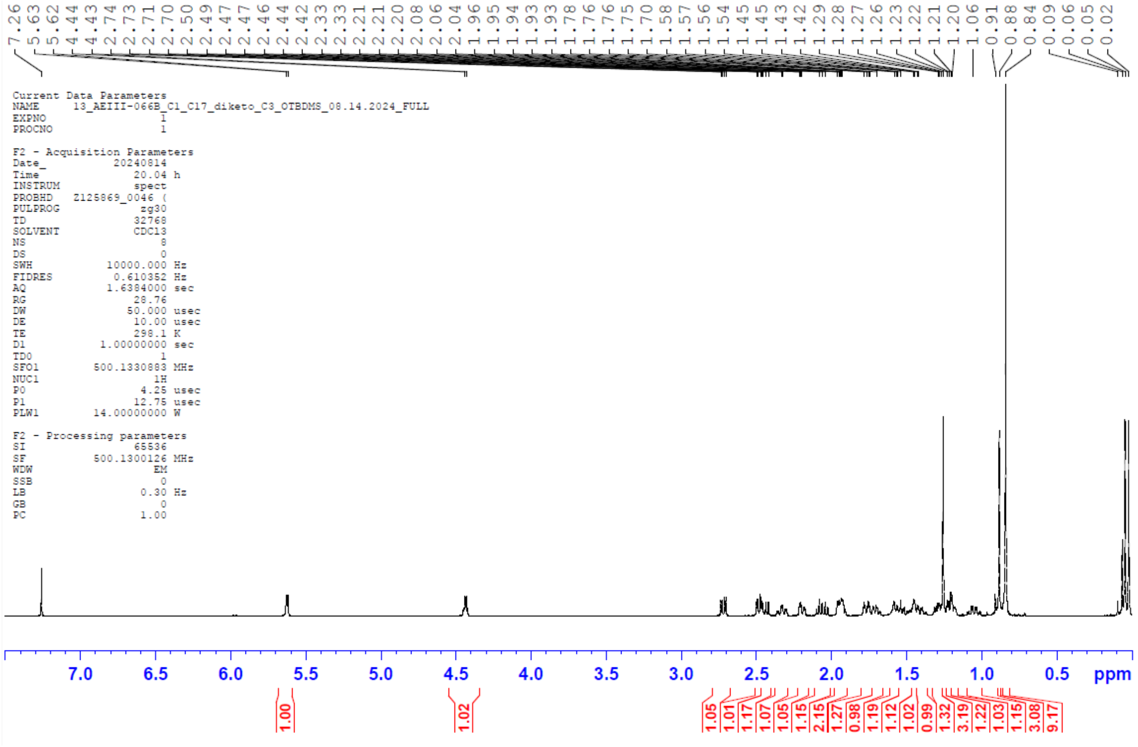

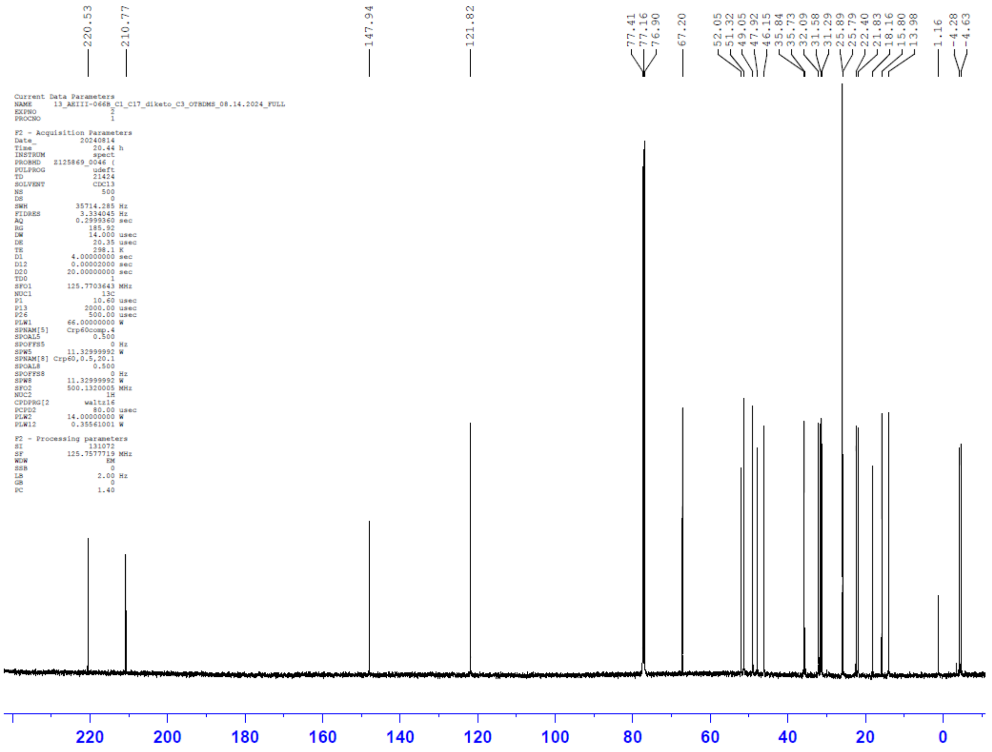

Figure S1.5-1. ^{1}H and ^{13}C NMR spectra (top and bottom) of compound **13**.

1.5. Step 5 (12 to 13): Oxidation of 1,17-Diol

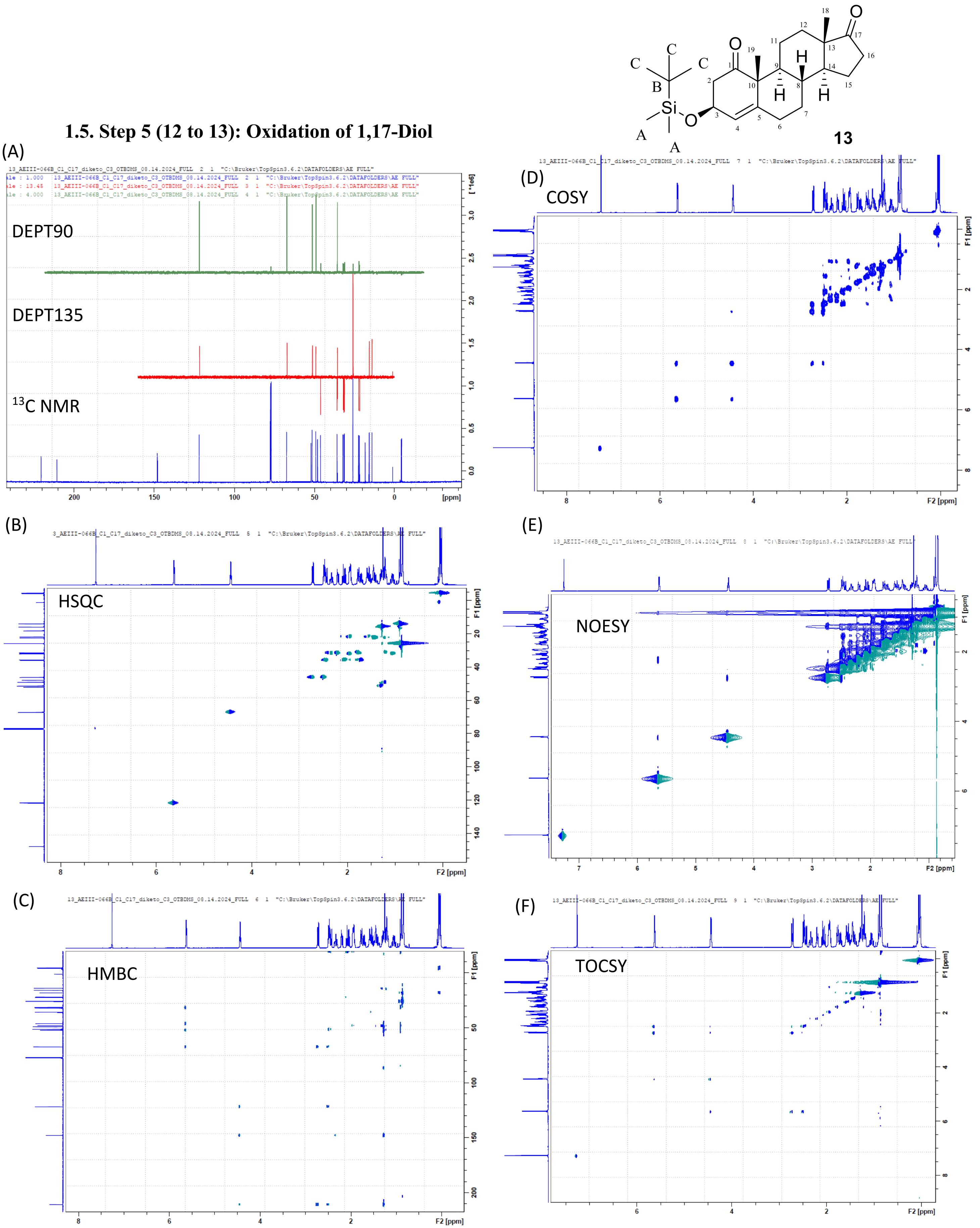

Figure S1.5-2: (A) DEPT90, DEPT135, ^{13}C NMR, (B) HSQC, (C) HMBC, (D) COSY, (E) NOESY, (F) TOCSY of 13.

1.5. Step 5 (12 to 13): Oxidation of 1,17-Diol

Table S1.5-1. NMR assignment of 3β-(*tert*-Butyldimethylsiloxy)-androst-4-en-1,17-dione (Compound **13**)

https://nmrxiv.org/S865

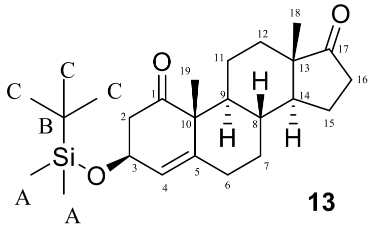

13

Position	^{13}C	^{1}H	Interactions
1(C=O)	210.5	--------	
2(-CH$_2$-)	46.22	2.70, 2.49	
3(-CH-)	67.4	4.439	4.439-HMBC-46.2 (C2), 121.4 (C4), 148.3 (C5), 211.3 (C1)
4(-CH=)	121.9	5.632	5.65-HMBC-67.2, 52.08, 46.22, 31.64 (2,5,6)
5	147.9	--------	
6(-CH$_2$-)	32.09	1.95	
7(-CH$_2$-)	31.3	2.20, 2.333	2.201-HMBC-35.8
8(-CH-)	51.32	1.289	1.289-HMBC-211 (C1), 148.3 (C5), 52.3 (), 49.4 (C9)
9(-CH-)	49.1	1.21	1.21-HMBC-14.1 (18), 22.5 (C12), 35.8 (C16), 48.2(C13), 51.5(C14)
10	52.08	--------	
11(-CH$_2$-)	31.5	1.033, 1.086	
12(-CH$_2$-)	21.8	1.394, 1.223	
13	47.92	--------	
14(-CH-)	51.32	1.294	
15(-CH$_2$-)	21.83	1.40, 1.23	
16(-CH$_2$-)	35.8	2.45, 2.06	
17(C=O)	220.7	--------	220.7-HMBC-2.47, 2.09, 1.99, 1.329, 0.918
18(-CH$_3$)	14.1	0.885	
19(-CH$_3$)	16.0	1.258	1.28-HMBC-148.2, 210.9, 49.1,51.98
A(Me)	25.89	0.862	
B(MeB)	1.21		
C(Si-C)	-4.39	0.09	

1.6. Step 6 (13 to 14): Stereoselective Reduction of 1,17-Diketone

Experimental Procedure for 3β-(*tert*-Butyldimethylsiloxy)-androst-4-en-1β,17β-diol (Compound **14**)

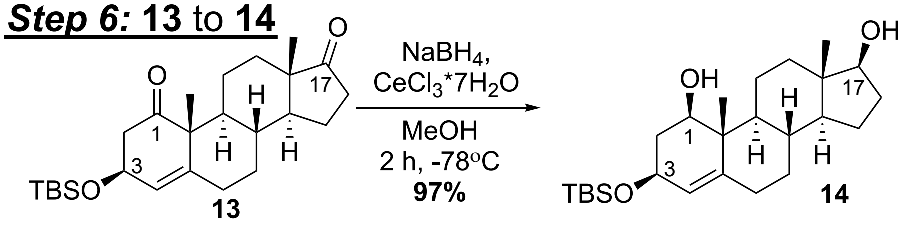

A solution of 3β-(*tert*-Butyldimethylsiloxy)-androst-4-en-1,17-dione (Compound **13**, 0.513 g, 1.25 mmol, 1.0 eq) and $CeCl_3 \cdot 7H_2O$ (0.932 g, 2.50 mmol, 2.0 eq) in MeOH (10 mL) was cooled to -78°C and left to stir for 15 minutes. $NaBH_4$ (0.095 g, 2.50 mmol, 2.0 eq) was added in small portions and the reaction was left stirring at -78°C for two hours. The reaction was washed with water (100 mL) and extracted with EtOAc (3 x 100 mL) to yield a white solid. The crude was directly loaded to a silica gel column chromatograph and purified (100% Hexanes to 100% Ethyl acetate) to afford 3β-(*tert*-Butyldimethylsiloxy)-androst-4-en-1β,17β-diol (Compound **14**) as white powdery solid (0.510 g, 1.212 mmol, 97%). mp: 120-125 °C. R_f = 0.634 (ethyl acetate:hexanes, 1:1, v/v). $[\alpha]^{20}_D$ = 0.028 [0.0052 g/10 mL in $(CH_3)_2CO$]; IR (neat) 2948.7, 2854.5, 1684.8, 1653.4, 1559.2, 1507.82, 1472.2, 1255.4, 835.9, 774.6 cm^{-1}; ^1H NMR (500 MHz, $CDCl_3$) 5.32 (s, 1H, H-4), 4.27 (s, 1H, H-3), 3.72 (m, 1H, H-1), 3.61 (t, J = 8.65 Hz, 1H, H-17), 2.32 (m, 1H, H-16), 2.07 (m, 1H, H-16), 2.02 (m, 1H, H-7), 1.91 (m, 1H, H-2), 1.84 (m, 1H, H-2), 1.80 (m, 1H, H-11), 1.79 (m, 1H, H-6), 1.76 (m, 1H, H-6), 1.60 (m, 1H, H-11), 1.57 (m, 1H, H-15), 1.52 (m, 1H, H-8), 1.46 (m, 1H, H-16), 1.41 (m, 1H, H-7), 1.30 (m, 1H, H-15), 1.23 (m, 1H, H-12), 1.13 (s, 3H, H-19), 1.07 (m, 1H, H-12), 1.01 (m, 1H, H-9), 0.91 (s, 9H, Si-$C(CH_3)_3$), 0.88 (m, 1H, H-14), 0.86 (m, 1H, H-9), 0.82 (m, 1H, H-9), 0.77 (s, 3H, H-18), 0.10 (s, 6H, $Si(CH_3)_2$); ^{13}C NMR (125 MHz, $CDCl_3$) 146.27, 122.74, 81.95, 73.51, 66.65, 54.08, 50.83, 43.75, 42.85, 37.35, 36.89, 36.30, 33.52, 32.47, 30.46, 26.02, 25.78, 23.58, 23.11, 18.31, 13.79, 11.16, 1.13, -4.50, -4.56. HRMS of **14** calculated for $[C_{25}H_{45}O_3Si]^+$: 421.3132, $[MH]^+$; found: 421.3126.

1.6. Step 6 (13 to 14): Stereoselective Reduction of 1,17-Diketone

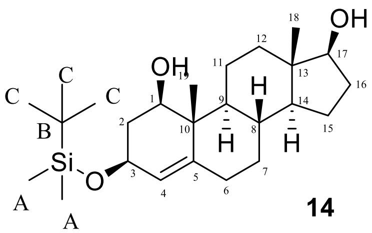

14

3β-(*tert*-Butyldimethylsiloxy)-androst-4-en-1β,17β-diol (Compound **14**)

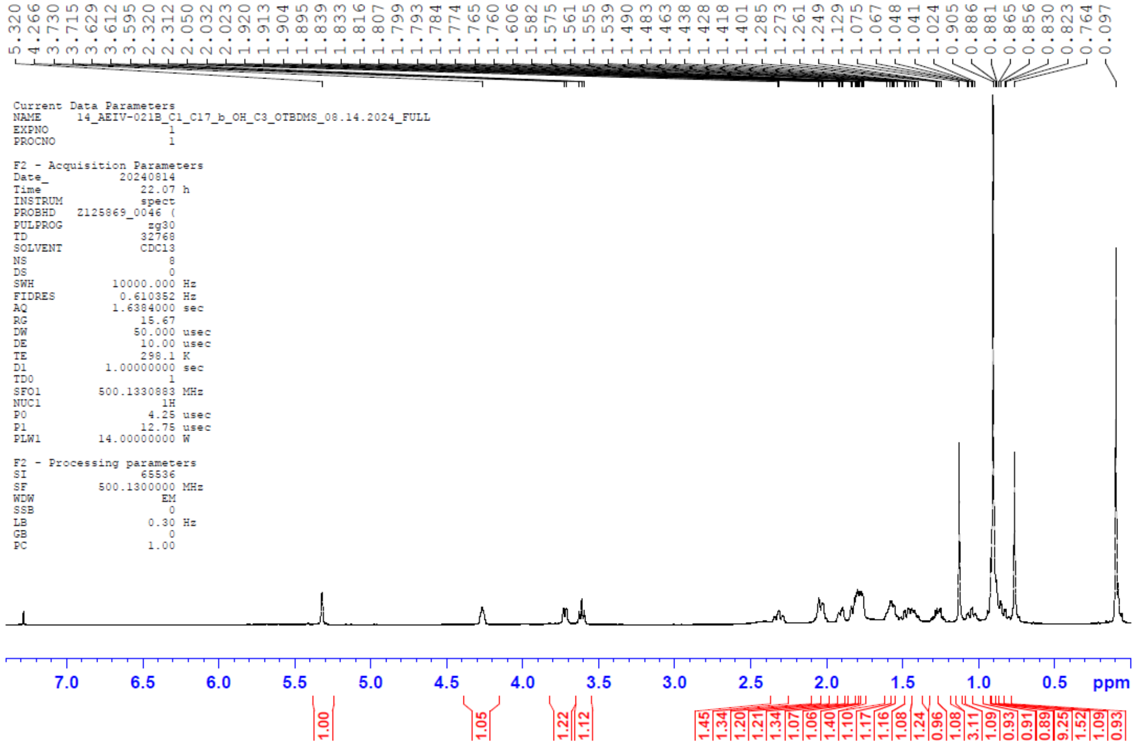

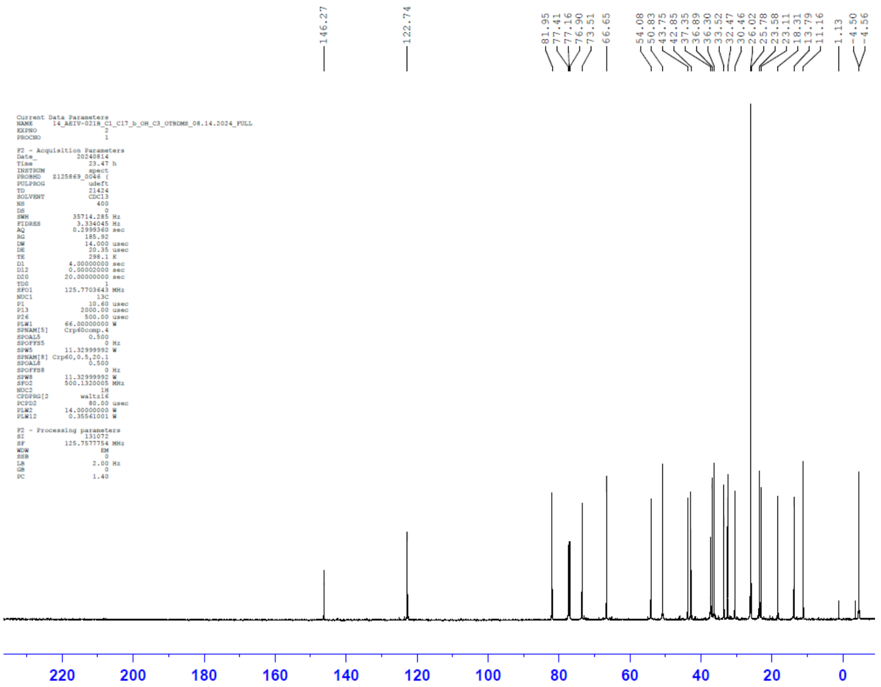

Figure S1.6-1. ^{1}H and ^{13}C NMR spectra (top and bottom) of compound **14**.

1.6. Step 6 (13 to 14): Stereoselective Reduction of 1,17-Diketone

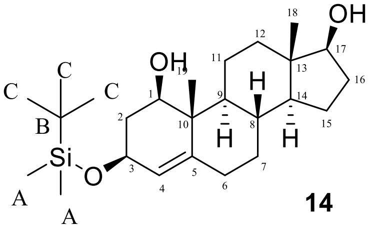

14

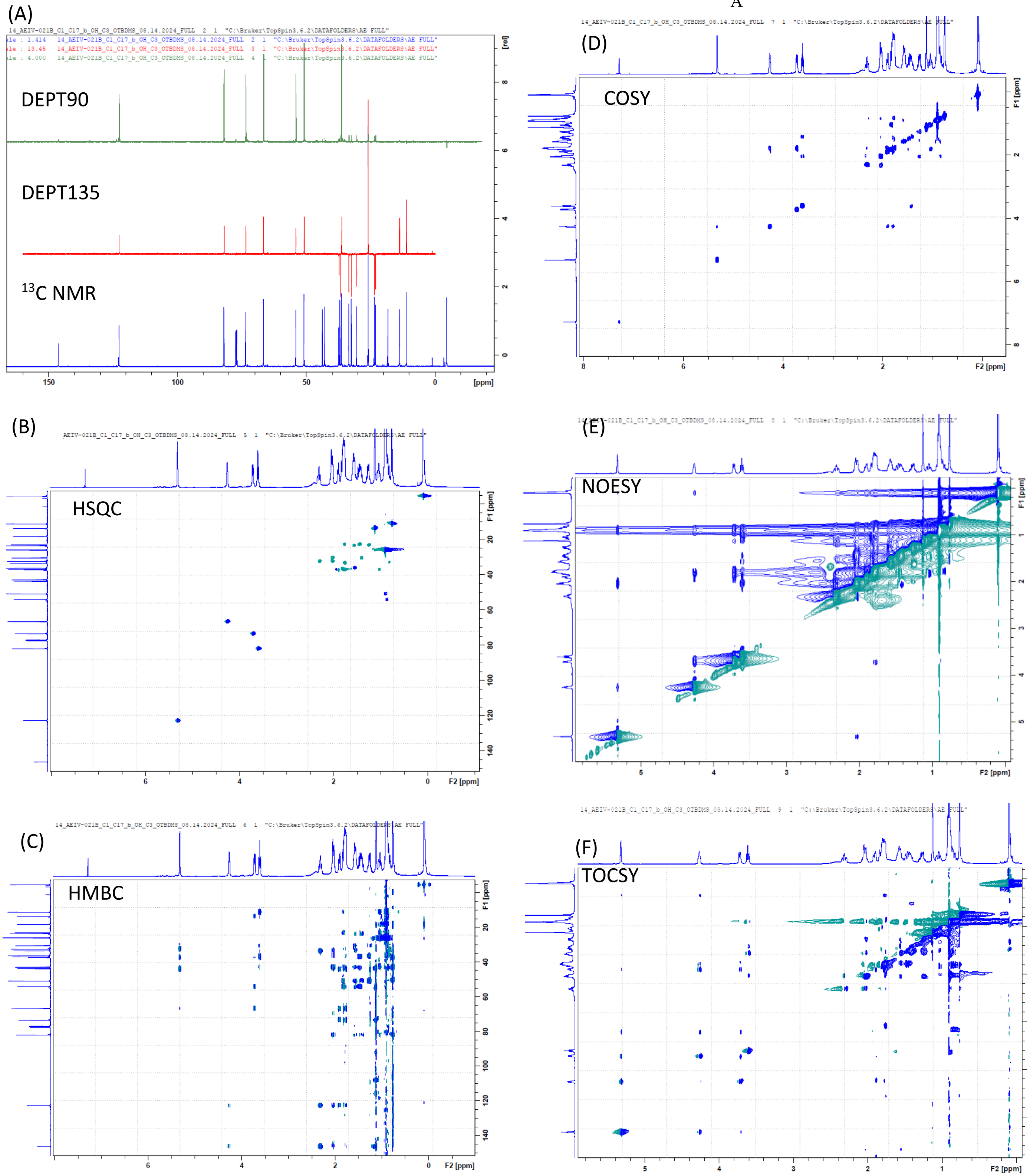

Figure S1.6-2: (A) DEPT90, DEPT135, ^{13}C NMR, (B) HSQC, (C) HMBC, (D) COSY, (E) NOESY, (F) TOCSY of 14.

1.6. Step 6 (13 to 14): Stereoselective Reduction of 1,17-Diketone

Table S1.6-1. NMR assignment of 3β-(*tert*-Butyldimethylsiloxy)-androst-4-en-1β,17β-diol (Compound **14**)

https://nmrxiv.org/S872

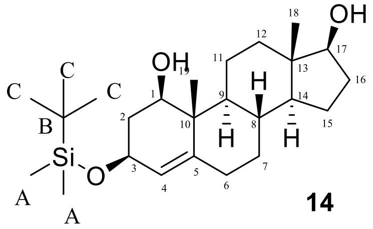

14

Position	^{13}C	^{1}H	Interactions
1(-CH-)	73.51	3.72	3.718-HMBC-1.1, 13.4(C19), 54.2, 66.7 (C3), 37.38
2(-CH2-)	37.38	1.90, 1.79	
3(-CH-)	66.67	4.27	4.266-HMBC- 36.88, 73.7, 122.82,146.3
4(-CH=)	122.74	5.32	5.34-HMBC-32.7, 37.32, 43.82
5	146.27	--------	
6(-CH2-)	33.52	1.76, 1.75	1.76-HMBC-66.6 (C3), 73.5 (C1), 122.9 (C4)
7(-CH2-)	30.4	2.02, 1.40	1.398-HMBC-23.58(C15), 50.9(C14)
8(-CH-)	36.29	1.56	1.56-HMBC-23.66 (C15), 30.4 (C7), 33.59 (C6), 50.7 (C14), 54.21 (C9)
9(-CH-)	54.2	0.87	
10	43.75	--------	
11(-CH2-)	23.11	1.76, 1.43	
12(-CH2-)	36.80	1.90, 1.04	
13	42.85	--------	
14(-CH-)	50.1	0.894	
15(-CH2-)	23.58	1.57, 1.25	1.25-HMBC-82.1(17),54.0(9),51.0(14),43.2(13),30.5(16)
16(-CH2-)	30.50	2.32, 2.07	
17(-CH-)	81.95	3.62	3.62-HMBC-11.4 (18), 36.8, 42.9 (13)
18(-CH3)	11.16	0.76	0.76-HMBC-43.0, 50.1, 82.2, 37.37 (18.6)
19(-CH3)	13.79	1.13	1.13-HMBC-43.64, 54.12, 73.28
A	-4.50, -4.56	0.096	
B	1.13		
C	26.02	0.90	

1.7. Step 7 (14 to 15): Deprotection of C3-TBS with TBAF

Experimental Procedure for Androst-4-en-1β,3β,17β-triol (Compound **15**)

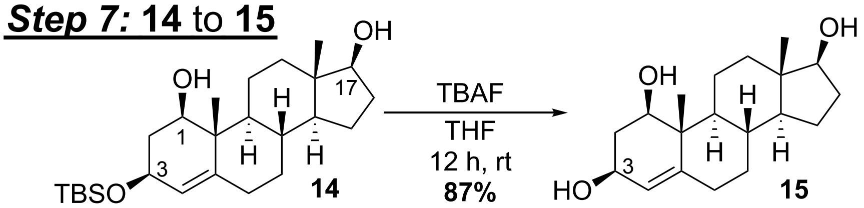

TBAF (403 μL, 1.391 mmol, 5.0 eq) was added to a solution of **14** (117 mg, 0.278 mmol, 1.0 eq) dissolved in molecular sieves dried THF (5 mL). The reaction was left stirring at RT overnight. The reaction was washed with water (100 mL) and extracted with EtOAc (3 x 100 mL) to yield a clear oil. The crude material was loaded to a silica gel column chromatograph and purified (100% Hexanes to 1:1 Ethyl acetate: methanol) to afford androst-4-en-1β,3β,17β-triol (compound **15**) as white solid (73.9 mg, 0.242 mmol, 87%). mp: 165-170 °C. R_f = 0.634 (100% ethyl acetate, v/v). $[\alpha]^{20}_D$ = 0.008 [0.0061 g/10 mL in MeOH]; IR (neat) 3328.2, 1650.5, 1085.6, 1044.24, 877.33 cm^{-1}; ^{1}H NMR (500 MHz, $CDCl_3$) 5.42 (s, 1H, H-4), 4.26 (s, 1H, H-3), 3.80 (m, 1H, H-1), 3.64 (t, J = 8.31 Hz, H-17), 2.33 (m, 1H, H-6), 2.11 (m, 1H, H-6), 2.07 (m, 1H, H-2), 2.03 (m, 1H, H-16), 1.87 (m, 1H, H-12), 1.84 (m, 1H, H-7), 1.81 (m, 1H, H-7), 1.78 (m, 1H, H-2), 1.75 (m, 1H, H-14), 1.52 (m, 1H, H-11), 1.47 (m, 1H, H-11), 1.43 (m, 1H, H-16), 1.33 (m, 1H, H-15), 1.16 (s, 3H, H-19), 1.08 (m, 1H, H-12), 0.97 (m, 1H, H-9), 0.88 (m, 1H, H-8), 0.78 (s, 3H, H-18); ^{13}CNMR (125 MHz, $CDCl_3$) 146.80, 133.93, 124.93, 122.32, 81.88, 73.65, 65.82, 54.08, 50.67, 43.56, 42.73, 37.35, 36.73, 36.19, 33.31, 32.31, 30.39, 23.48, 23.10, 13.52, 11.04, 1.03. HRMS of **15** calculated for $[C_{19}H_{29}O_3]^+$: 305.2111, $[MH-2H]^+$; found: 305.2109. Androst-4-en-1β,3β,17β-triol (Compound **15**) was crystallized through slow evaporation in methanol. CCDC deposit number of **15**: 2448101.

1.7. Step 7 (14 to 15): Deprotection of C3-TBS with TBAF

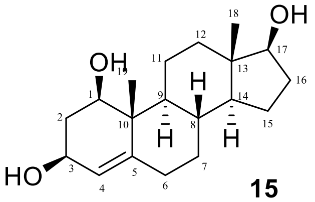

15

Androst-4-en-1β,3β,17β-triol (Compound **15**)

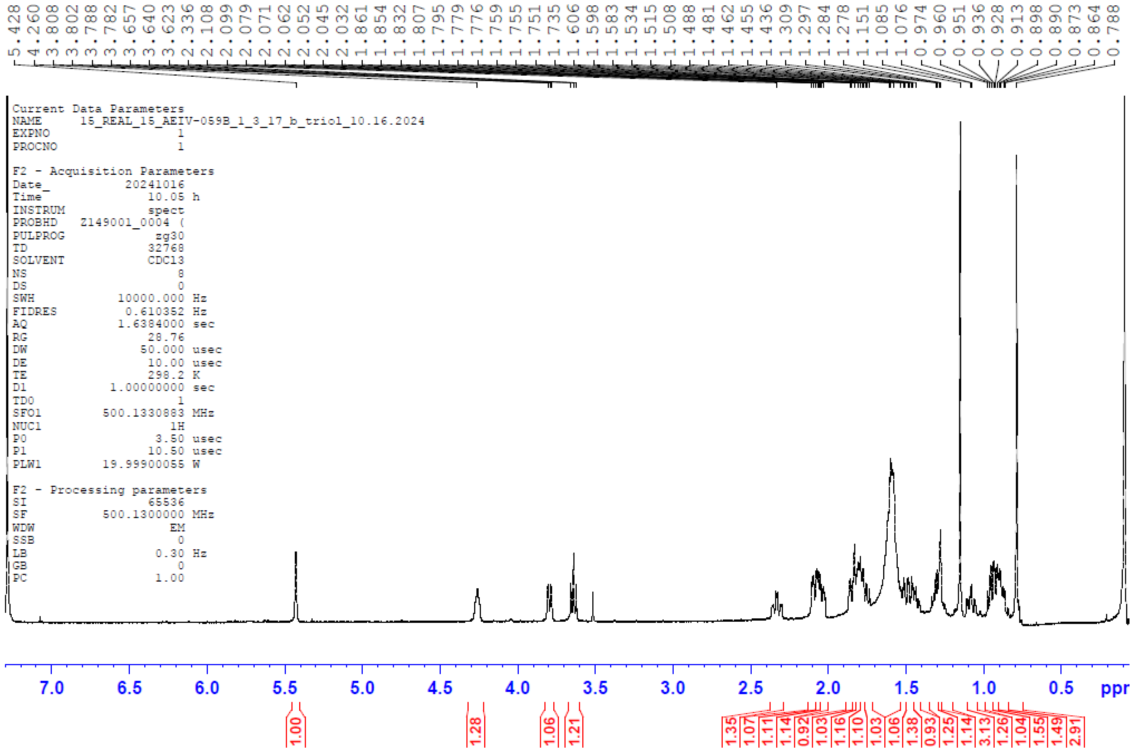

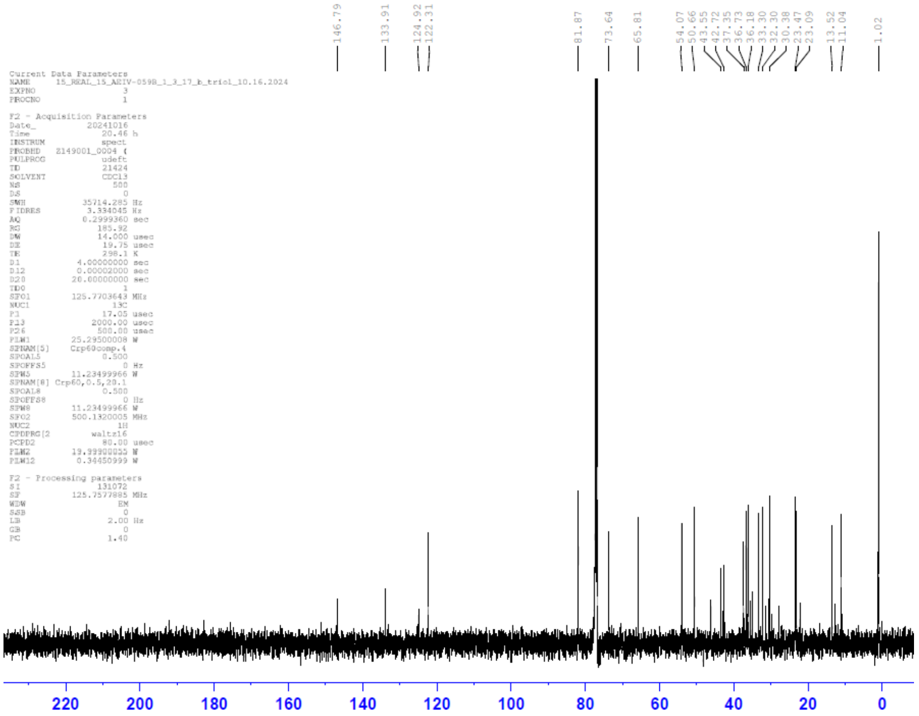

Figure S1.7-1. ^{1}H and ^{13}C NMR spectra (top and bottom) of compound **15**.

1.7. Step 7 (14 to 15): Deprotection of C3-TBS with TBAF

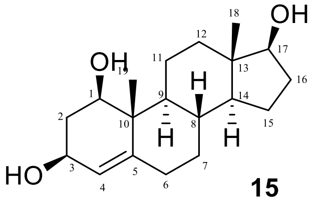

15

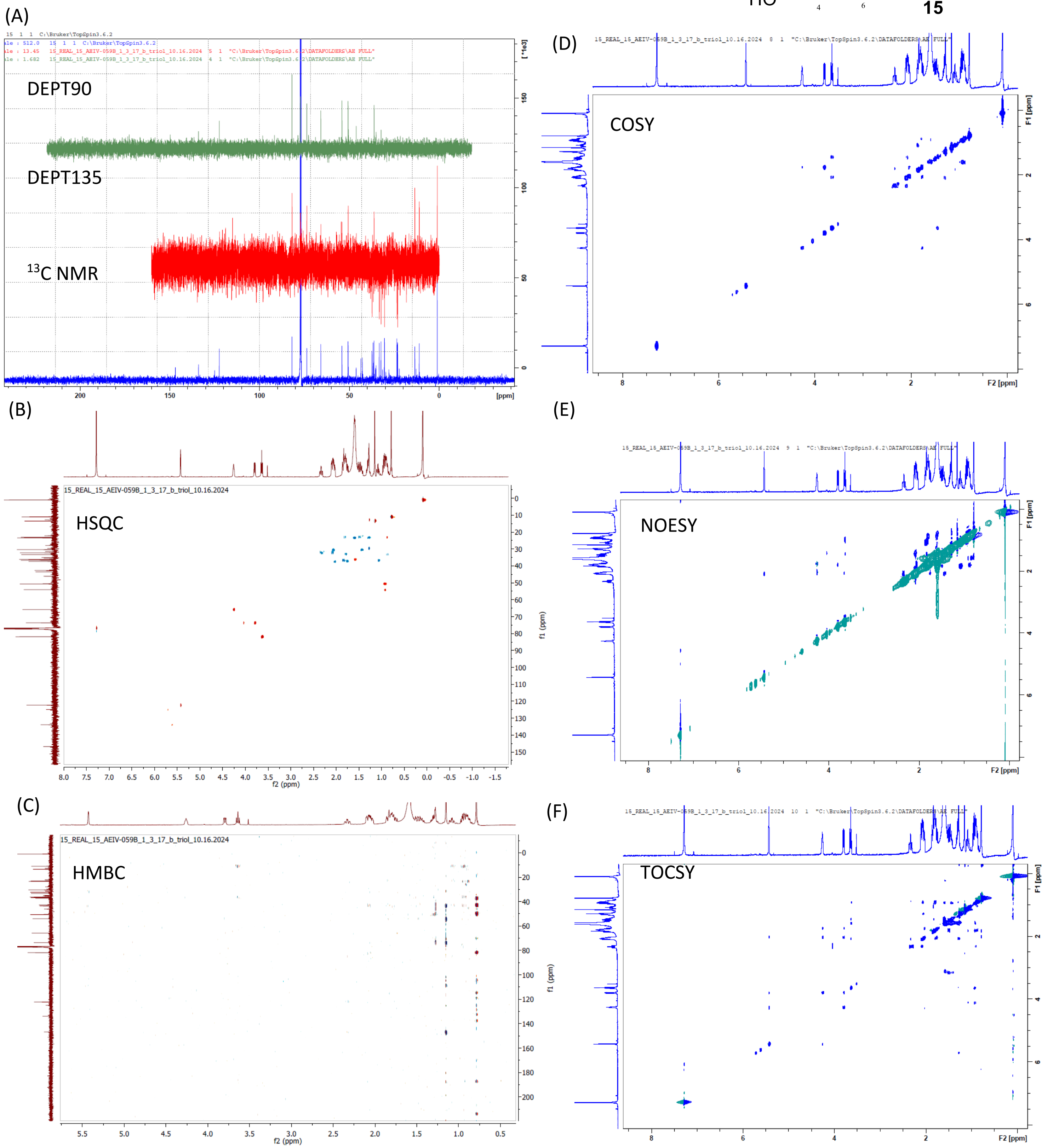

Figure S1.7-2: (A) DEPT90, DEPT135, ^{13}C NMR, (B) HSQC, (C) HMBC, (D) COSY, (E) NOESY, (F) TOCSY of 15.

1.7. Step 7 (14 to 15): Deprotection of C3-TBS with TBAF

Table S1.7-1. NMR assignment of Androst-4-en-1β,3β,17β-triol (Compound **15**)

https://nmrxiv.org/S854

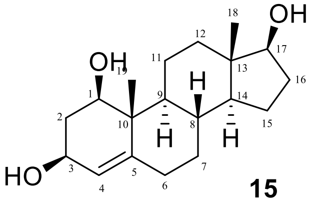

15

Position	^{13}C	^{1}H	Interactions
1(-CH-)	73.7	3.79	3.79- HMBC-54.2 (CH), 1.4, 13.5, 65.6
			3.79-TOCSY-1.74, 2.04
2(-CH2-)	37.3	1.76, 2.04	
3(-CH-)	65.82	4.258	4.26- HMBC-4.26, 4.26-COSY-2.04(C2)
			4.26-TOCSY-2.02, 1.76
4(-CH=)	122.4	5.436	5.436- HMBC- 43.64 (C), 32.51
5	146.8		
6(-CH2-)	32.4	2.1, 2.32	2.32-HMBC-121, 147
			2.32-COSY-1.80
7(-CH2-)	23.0	1.83, 1.80	
			1.79-COSY-0.88
8(-CH-)	23.2	0.88	
9(-CH-)	54.4	0.95	54.4-HMBC-1.16
10	43.6 (Rule Out)		43.6-HMBC-1.16
11(-CH2-)	22.9	1.48, 1.51	
12(-CH2-)	37.35	1.07, 1.84	1.84-COSY-1.07, 1.51, 1.81-TOCSY-1.49, 1.51, 1.55, 1.57, 1.46, 1.42
13	42.9		42.9-HMBC-1.15, 1.28, 2.06
14(-CH-)	36.0	1.75	
15(-CH2-)	23.4	1.59, 1.30	1.59-TOCSY-3.64
16(-CH2-)	30.4	1.43, 2.04	1.43,2.04-COSY-3.64,
			2.04-COSY-1.77 (37.4-HSQC-1.77, 2.02)
17(-CH-)	81.8	3.64	3.64-HMBC-11.0, 36.21 (CH), 3.64-COSY-2.04, 1.43(C16)
			3.64-TOCSY-1.45, 1.59, 2.08
18(-CH3)	11.0	0.79	0.79-HMBC-82.1 (17), 50.7 (CH), 42.9(C)(C13), 37.35(CH2)(C12)
19(-CH3)	13.6	1.16	1.16-HMBC-147.3, 73.9, 54.4, 43.58

1.8. Step 8 (15 to 2): Regioselective Oxidation of Triol 15

Experimental Procedure for 1β-Hydroxytestosterone (Compound **2**)

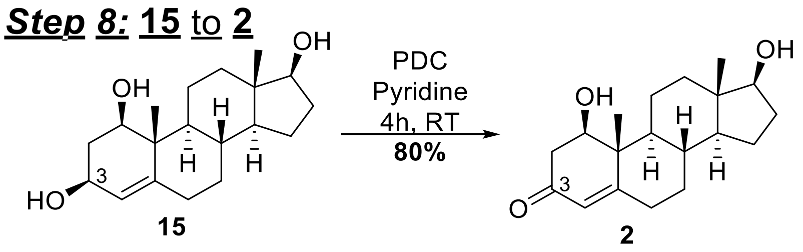

PDC (61.5 mg, 0.164 mmol, 1.0 eq) was added to a solution of androst-4-en-1β,3β,17β-triol (Compound **15**, 50.0 mg, 0.164 mmol, 1.0 eq) dissolved in molecular sieves dried pyridine (6.0 mL). The reaction was left stirring at RT for four hours. The reaction was washed with water (100 mL) and extracted with EtOAc (3 x 100 mL). The crude was loaded to a silica gel column chromatograph and purified (100% Hexanes to 1:1 Ethyl acetate: methanol) to afford 1β-hydroxytestosterone (Compound **2**) as a white solid (39.9 mg, 0.131 mmol, 80%). mp of **2**: 155-160 ℃. Literature value of mp of **2**: 174-175 ℃.[4] R_f = 0.636 (100% ethyl acetate, v/v). $[\alpha]^{20}_D$ = 0.001 [0.0061 g/10 mL in MeOH]; IR (neat) 3395.2, 2925.9, 2870.2, 1654.8, 1612.0, 1355.2, 1291.0, 1067.1, 1042.8, 734.7, 701.9 cm^{-1}; ^1H NMR (500 MHz, $CDCl_3$) 5.79 (s, 1H, H-4), 4.04 (t, J = 7.62 Hz, 1H, H-1), 3.64 (t, J = 8.50 Hz, 1H, H-17), 2.54 (m, 2H, H-2), 2.48 (m, 1H, H-6), 2.32 (m, 1H, H-6), 2.07 (m, 1H, H-16), 1.99 (m, 1H, H-11), 1.89 (m, 1H, H-7), 1.84 (m, 1H, H-12), 1.67 (m, 1H, H-7), 1.63 (m, 1H, H-8), 1.60 (m, 1H, H-11), 1.48 (m, 1H, H-16), 1.42 (m, 1H, H-15), 1.31 (m, 1H, H-15), 1.25 (s, 3H, H-19), 1.15 (m, 1H, H-12), 1.05 (m, 1H, H-7), 0.99 (m, 1H, H-14), 0.80 (s, 3H, H-18); ^{13}CNMR (125 MHz, $CDCl_3$) 197.31, 170.17, 123.66, 81.71, 74.09, 54.41, 50.43, 44.96, 43.72, 42.64, 36.65, 36.15, 33.39, 32.37, 30.33, 23.47, 23.44, 12.54, 11.04.

4. P.K. Sharma, A. Akhila, *Indian J. Chem.* 1991**,** *30B*, 554-556.

1.8. Step 8 (15 to 2): Regioselective Oxidation of Triol 15

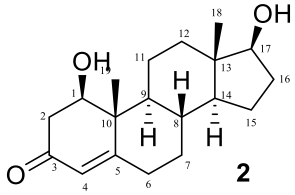

2

1β-Hydroxytestosterone (Compound **2**)

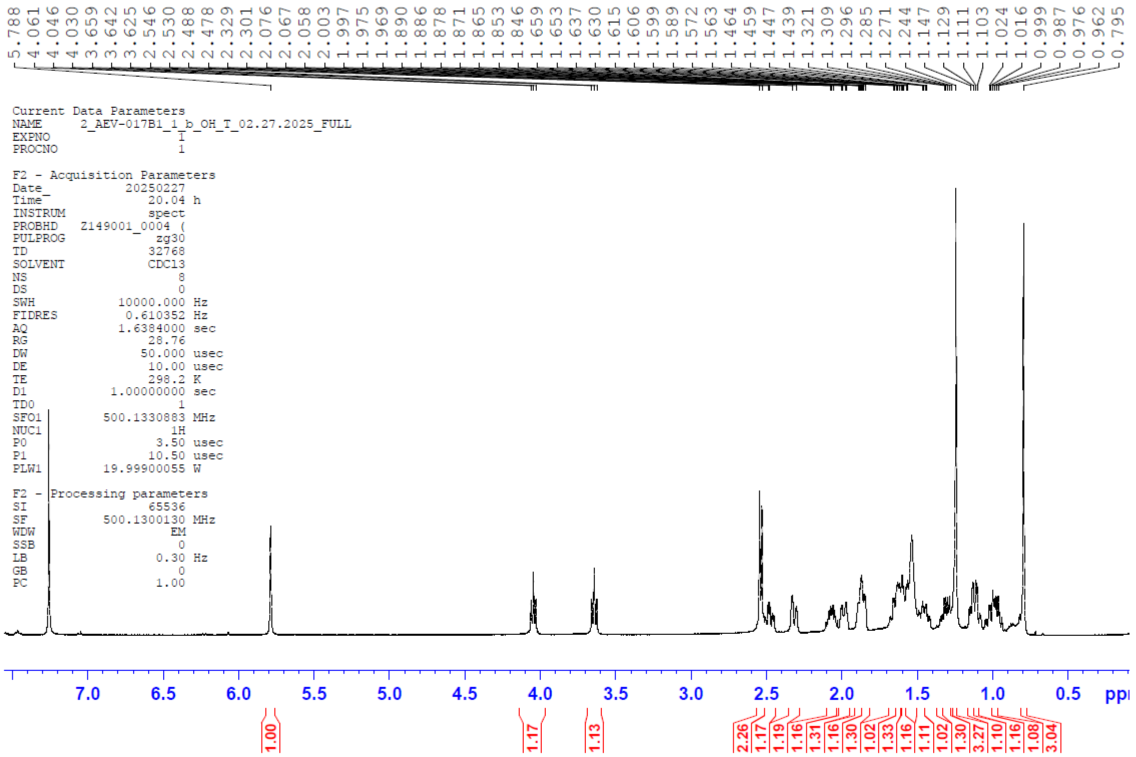

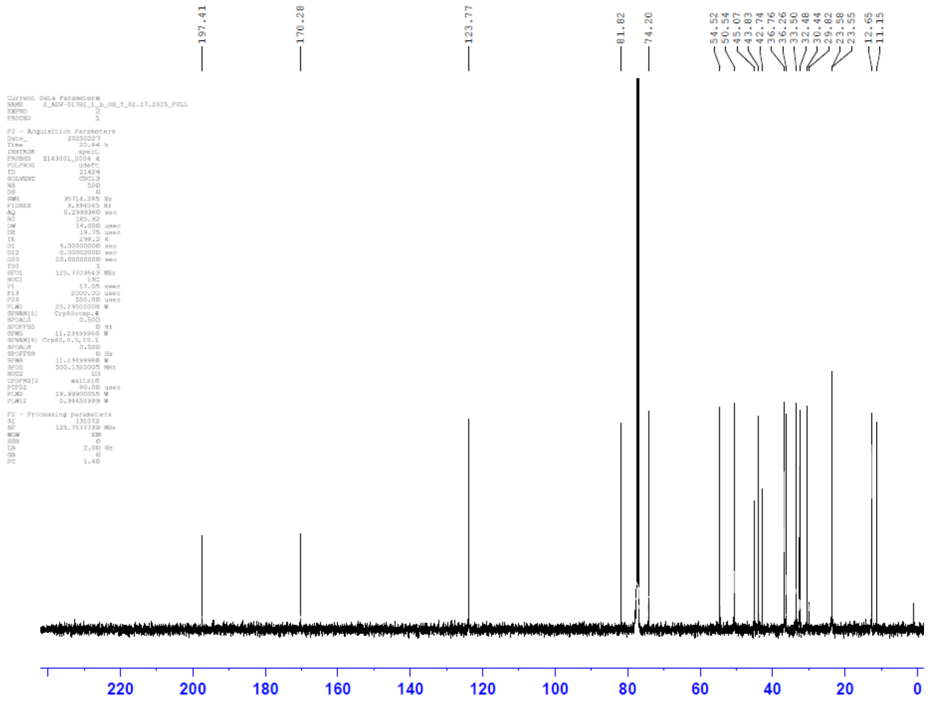

Figure S1.8-1. ^{1}H and ^{13}C NMR spectra (top and bottom) of compound **2**.

1.8. Step 8 (15 to 2): Regioselective Oxidation of Triol 15

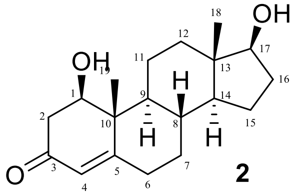

2

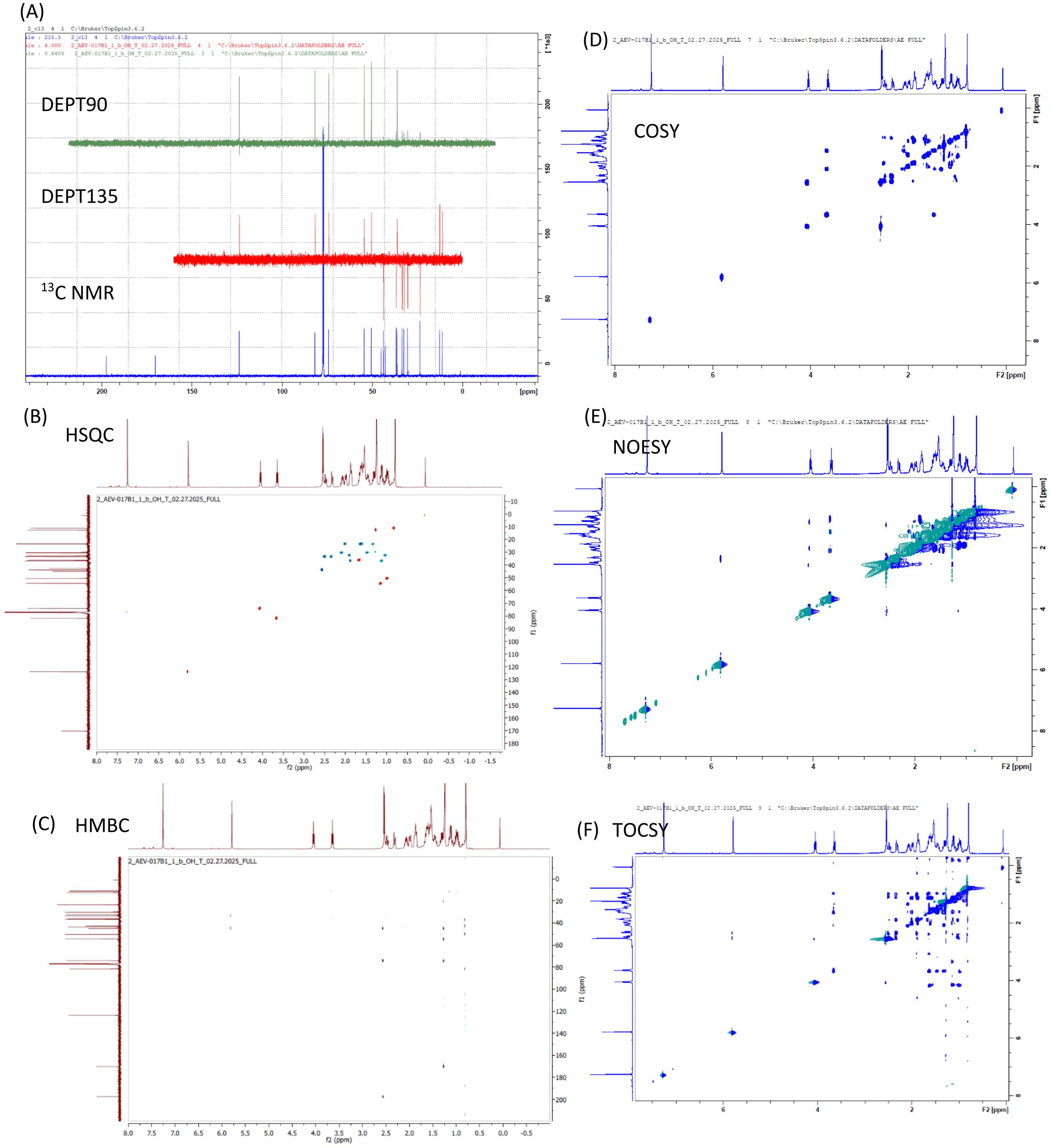

Figure S1.8-2: (A) DEPT90, DEPT135, ^{13}C NMR, (B) HSQC, (C) HMBC, (D) COSY, (E) NOESY, (F) TOCSY of 2.

1.8. Step 8 (15 to 2): Regioselective Oxidation of Triol 15

Table S1.8-1. NMR assignment of 1β-Hydroxytestosterone (Compound **2**)

https://nmrxiv.org/S866

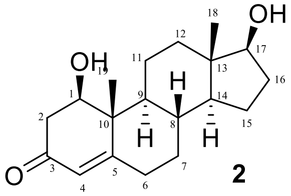

2

Position	^{13}C	^{1}H	Interactions	multiplicity
1	74.1	4.074	4.07-COSY-2.59	triplet, J = 7.9 Hz
2	4.39	2.59		
3	197.3			
4	123.79	5.81	5.72-HMBC-168.51, 124-HMBC-2.51	
5	168.51		168-HMBC-2.51	
6	33.3	2.51, 2.34		
7	32.5	1.90, 1.04	through elimination (DEPT135: -CH_2-)	
8	37.0	1.67	(through elimination)	
9	54.6	1.15	54.6-HMBC-1.23(C19)	
10	44.9		1.23-HMBC-44.9	
11	23.5	2.00, 1.63	23.5-HMBC-1.15, 2.00-COSY-1.15	
12	36.8	1.89, 1.14		
13	42.8			
14	49.9	1.00	49.9-HMBC-0.83	
15	23.7	1.42, 1.33		
16	30.5	2.07, 1.48	2.07-COSY-1.33	
17	81.66	3.68	3.68-COSY-2.07, 1.48	triplet, J = 8.3 Hz
			81.7-HMBC-2.10 (C16), 1.14 C12), 0.83 (C18)	
18	11.0	0.83	0.83-HMBC-42.8 (C13)	
			0.83-HMBC-49.9 (-CH-, 49.9 has 1.0 ppm proton)	
19	12.7	1.28	1.25-HMBC-54.6(C9)	

1.9. Synthesis of 1α-Hydroxytestosterone (Compound 9)

Experimental Procedure for 1α-Hydroxytestosterone (Compound 9)

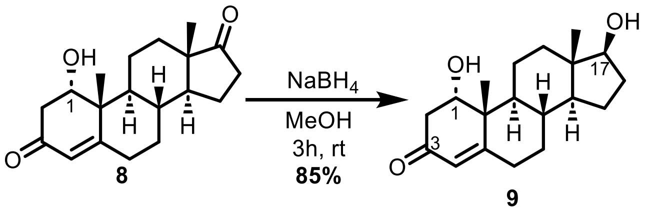

1α-Hydroxy-androst-4-en-3,17-dione (Compound **8**, 0.148 g, 0.489 mmol, 1.0 eq) was dissolved in MeOH (15 mL) and $NaBH_4$ (0.19 g, 0.489 mmol, 1.0 eq) was added. The reaction was stirred at rt for 3 h. The reaction was washed with H_2O (25 mL) and extracted with EtOAc (3 x 25 mL). The organic layer was concentrated under reduced pressure to yield an oil. The crude was purified via silica gel column chromatography (1:4 EtOAc in hexanes to 1:1 EtOAc in MeOH) to give 1α-hydroxytestosterone (Compound **9**) as a white solid (0.120 g, 0.414 mmol, 85%). mp of **9**: 180-188 °C. Literature value for mp of **9**: 180-182 °C.[2] R_f = 0.350 (100% EtOAc, v/v). $[\alpha]^{20}_D$ = -0.045 [0.0026 g/10 mL in MeOH]; IR (neat) 3395.20, 2938.7, 2921.58, 1653.38, 1262.5, 1058.50, 764.63, 750.37 cm^{-1}; ^1H NMR (500 MHz, d_6-DMSO) 5.59 (s, 1H, H-4), 3.88 (s, 1H, H-1), 3.43 (t, J = 8.75 Hz, 1H, H-17), 2.66 (dd, J_1 = 16.50 Hz, J_2 = 1.95 Hz, H-2), 2.35 (m, 1H, H-6), 2.29 (m, 1H, H-2), 2.23 (m, 1H, H-6), 1.83 (m, 1H, H-16), 1.73 (m, 1H, H-12), 1.67 (m, 1H, H-7), 1.56 (m, 1H, H-8), 1.50 (m, 1H, H-11), 1.46 (m, 1H, H-15), 1.34 (m, 1H, H-16), 1.28 (m, 1H, H-15), 1.20 (m, 1H, H-11), 1.09 (s, 3H, H-19), 0.98 (m, 1H, H-12), 0.91 (m, 1H, C9), 0.83 (m, 1H, H-7), 0.66 (s, 3H, H-18); ^{13}C NMR (125 MHz, d_6-DMSO) 198.13, 168.25 122.86, 80.41, 70.77, 50.64, 44.76, 43.12, 43.03, 42.80, 36.65, 35.18, 32.69, 30.89, 29.98, 23.51, 19.84, 18.51, 11.62. HRMS of **9** (HESI) calculated for $[C_{19}H_{29}O_3]^+$: 305.2111, $[MH]^+$; found: 305.2110. 1α-Hydroxytestosterone (**9**) was crystallized through slow evaporation of 100% EtOAc. CCDC deposit # of **9**: 2448104.

2. B. Pelc, J. Hodkova, *Collect. Czech. Chem. Commun.*, 1967, 32, 410-418.

1.9. Synthesis of 1α-Hydroxytestosterone (Compound 9)

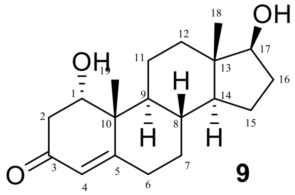

9

1α-Hydroxytestosterone (Compound **9**)

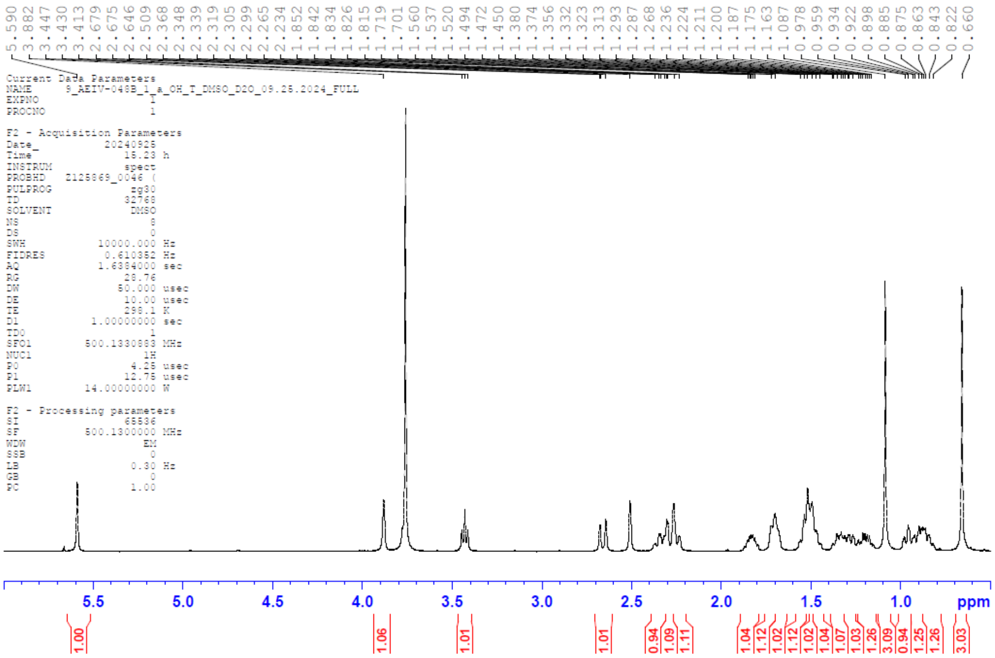

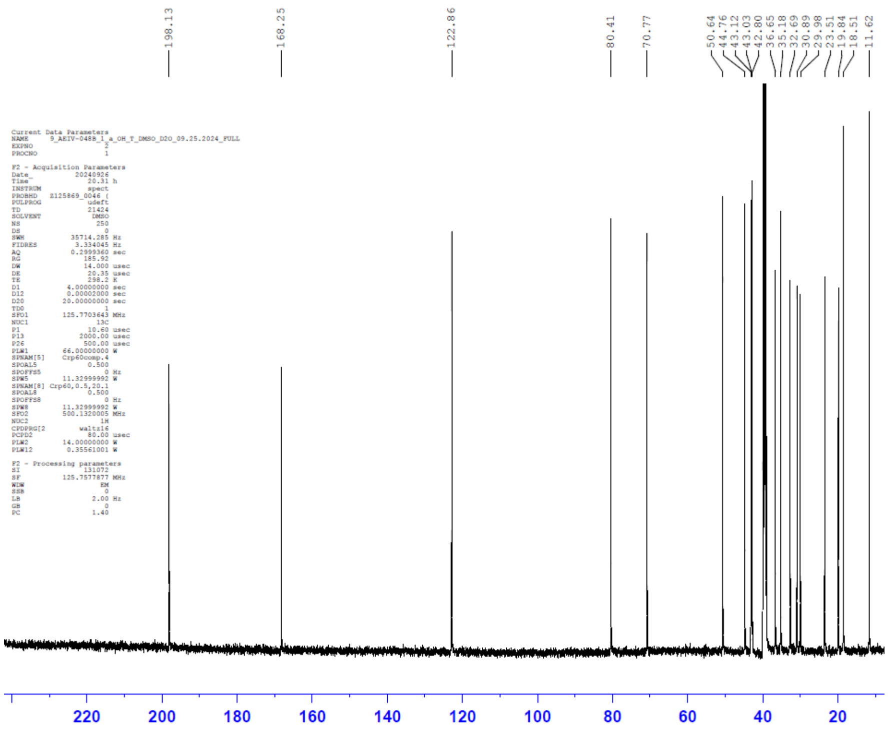

Figure S1.9-1. ^{1}H and ^{13}C NMR spectra (top and bottom) of compound **9**.

1.9. Synthesis of 1α-Hydroxytestosterone (Compound 9)

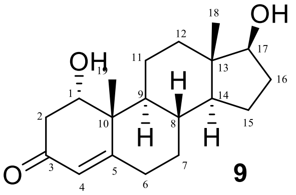

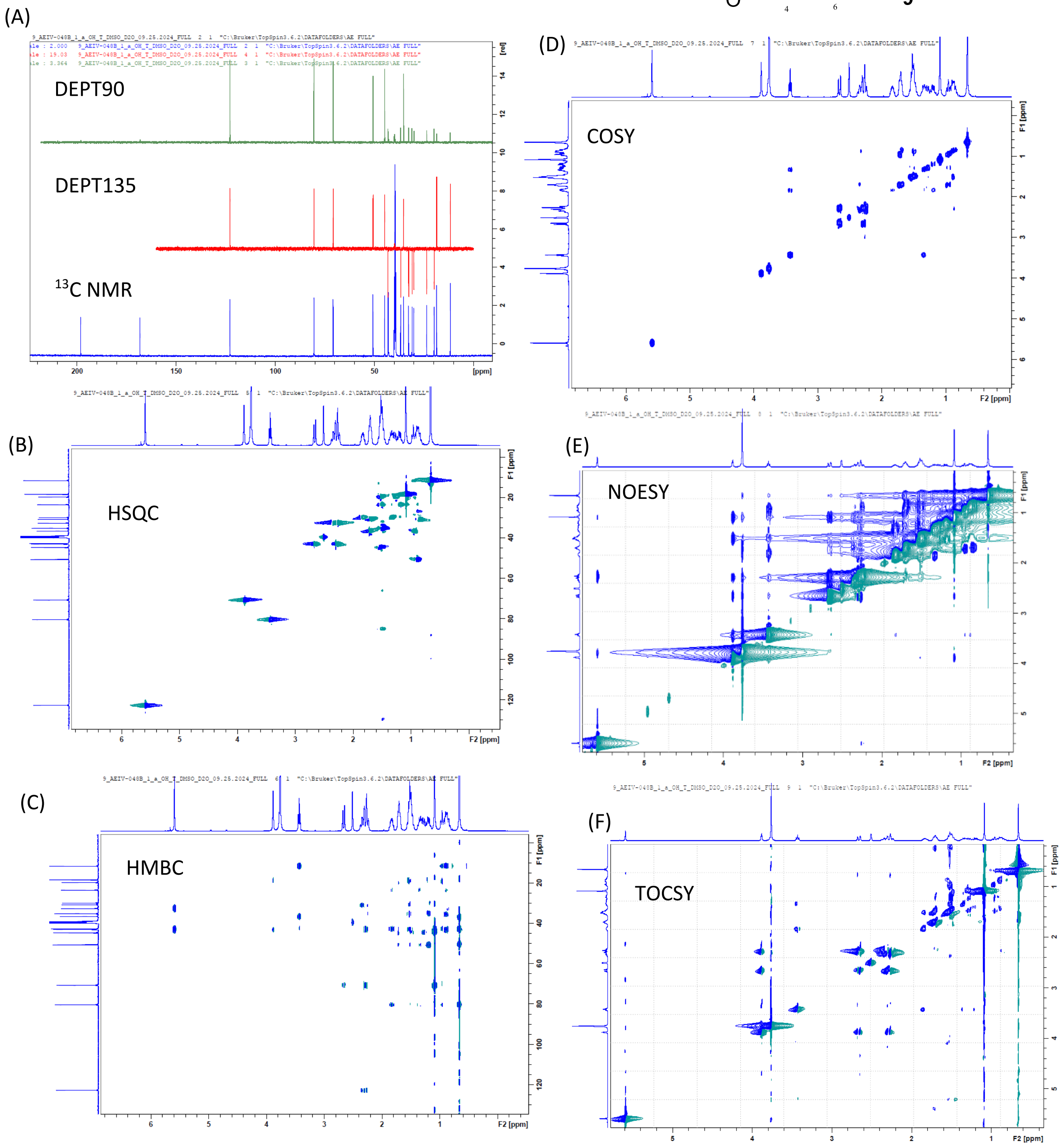

Figure S1.9-2: (A) DEPT90, DEPT135, ^{13}C NMR, (B) HSQC, (C) HMBC, (D) COSY, (E) NOESY, (F) TOCSY of 9.

1.9. Synthesis of 1α-Hydroxytestosterone (Compound 9)

Table S1.9-1. NMR assignment of 1α-Hydroxytestosterone (**Compound 9**)

https://nmrxiv.org/S864

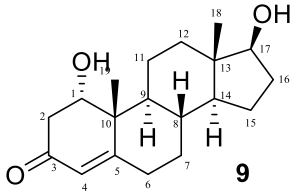

9

Position	^{13}C	^{1}H	Interactions
1(-CH-)	70.6	3.87	
2(-CH₂-)	43.17	2.65, 2.28	
3	198.5		198.5-HMBC-2.30(C2), 2.67(C2), 3.90(C1)
4(-CH=)	123.1	5.59	5.59-HMBC-43.0, 32.5
			123-HMBC-2.33
5	168.8		168.8-HMBC-3.91, 2.35, 1.08, 1.69
6(-CH₂-)	32.4 (C6)	2.33, 2.24	2.24, 2.33-COSY-0.86(C7)
7(-CH₂-)	31.0	0.85, 1.68	1.70-TOCSY-1.46, 1.68-COSYH-0.86
8(-CH-)	44.8	1.52	
9(-CH-)	50.7	0.88	
10		43.0	(Rule out using ^{13}C and DEPT90, DEPT135)
11(-CH₂-)	23.5	1.17, 1.50	(DEPT135 RULE OUT)
12(-CH₂-)	36.9	0.94, 1.70	
13	42.7		42.7-HMBC-0.66
14(-CH-)	0.87	50.5	
15(-CH₂-)	19.5	1.51, 1.26	
16(-CH₂-)	29.8	1.82, 1.33	1.89-COSY-3.42
			1.83-COSY-1.22
17(-CH-)	80.3	3.42	3.42-COSY-1.89, 1.34
18(-CH₃)	11.6	0.66	0.66-HMBC-80.4(C17), 50.0(C14, DEPT90), 42.7(C13), 36.9(C12)
19(-CH₃)	18.2	1.09	1.09-HMBC-169.7, 71.2, 43.6

NMR was taken in d_6-DMSO with a drop of D_2O (to exchange the O-H protons), referenced to δ 2.50 (^{1}H) and 39.52 (^{13}C).

2. Optimization of Step 1: 1,4-Borylation (Table 1 from Main Text)

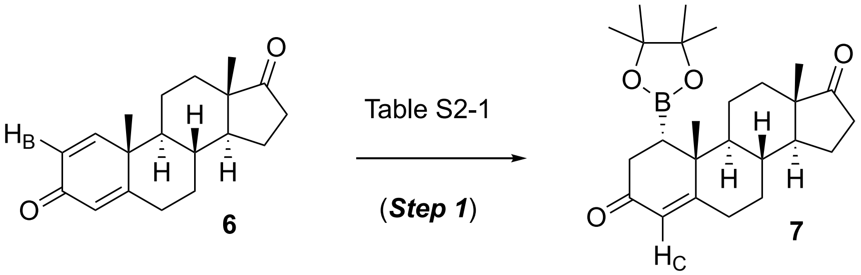

Figure S2-1. Optimization of the C1-borylation of androst-1,4-dien-3,17-dione (**6** to **7**) (Table S2-1).

During the optimization of the borylation reaction at C1 of androst-1,4-dien-3,17-dione, we considered a variety of reaction conditions (Table S2-1, entries 1-15) to ultimately find that DBU-promoted formation of the carbene ligand for the copper catalyst was ideal. A phosphine-promoted hydroxylation of an activated olefin was tested (entries 1-2),[1] Rhodium-catalyzed conditions with a phosphine ligand was considered (entries 1-2),[2, 3] as well as copper with potassium *tert*-butoxide in the presence of the imidazolinium reagent to form a carbene in situ (entries 3-8),[4-6] in addition, the use of methanol instead of copper bromide was also tested (entries 9-11). In the end, the DBU-promoted carbene formation was the optimal condition to conduct the 1,4-borylation of the substrate (entries 12-15).

2. Optimization of Step 1: 1,4-Borylation (Table 1 from Main Text)

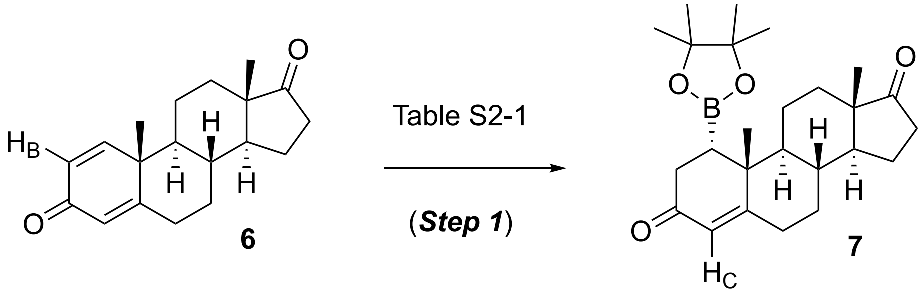

Table S2-1. Optimization of C1-borylation of androst-1,4-dien-3,17-dione (Entries 1-8).

Entry	Conditions	Result (Conversion)[a]
**1**	AD (1.0 eq), $(PinB)_2$ (1.0 eq), TPP (0.76 eq), MeOH (0.07 M), Wilkinson's Catalyst (0.3 eq)	17.9%[a]
**2**	AD (1.0 eq), $(PinB)_2$ (1.1 eq), DBU (45 eq), Imidazolinium (45 eq), THF (0.07 M)<br>(KOtBu was replaced with DBU and no CuBr was added to the reaction)	No desired product
**3**	AD (1.0 eq), $(PinB)_2$ (3.5 eq), CuBr (9.3 eq), KOtBu (7.1 eq), Imidazolinium (4.2 eq), THF (0.8 M)<br>(Copper complex was made in a neat fashion and was added after 5 minutes)	39.9%[a]
**4**	AD (1.0 eq), $(PinB)_2$ (1.3 eq), CuBr (0.2 eq), DBU (1.8 eq), Imidazolinium (1.8 eq), THF (1.8 M)<br>(0.2 eq of CuBr was used in the reaction)	74.5%[a]
**5**	AD (1.0 eq), $(PinB)_2$ (1.5 eq), CuBr (0.5 eq), DBU (1.0 eq), Imidazolinium (1.0 eq),<br>1,4-dioxane (molecular sieves dried) (1.0 M)	94.0%[a]

[a]: The percent conversion to the borylation product was calculated by integrating the proton signal at δ 6.2 ppm of the C2-proton of androstdiendione (starting material) and the signal at δ 5.8 ppm of the C4 proton of the C1-borane product. The imidazolinium reagent in entries 3-16 used was: [EMIM][ESO_4]: 1-ethyl-3-methylimidazolinium ethyl sulfate. TPP: triphenylphosphine, AD: androstdiendione.

2. Optimization of Step 1: 1,4-Borylation (Table 1 from Main Text)

To illustrate the optimization process of the borylation of boldione, the following 3 figures (Figure S2-2) show the ^{1}H NMR spectra of the crude reaction mixtures of Table S2-1 (also see Table 1 in the main text). Shown in the overlay are the signals for H_B (position-2, δ 6.2) of boldione (compound **6**) and H_C (position-4, δ 5.8) of the borylated product, 1α-pinacolatoboryl-androst-4-en-3,17-dione (compound **7**).

(A)

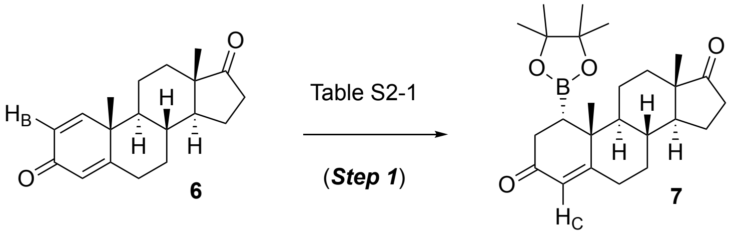

(B)

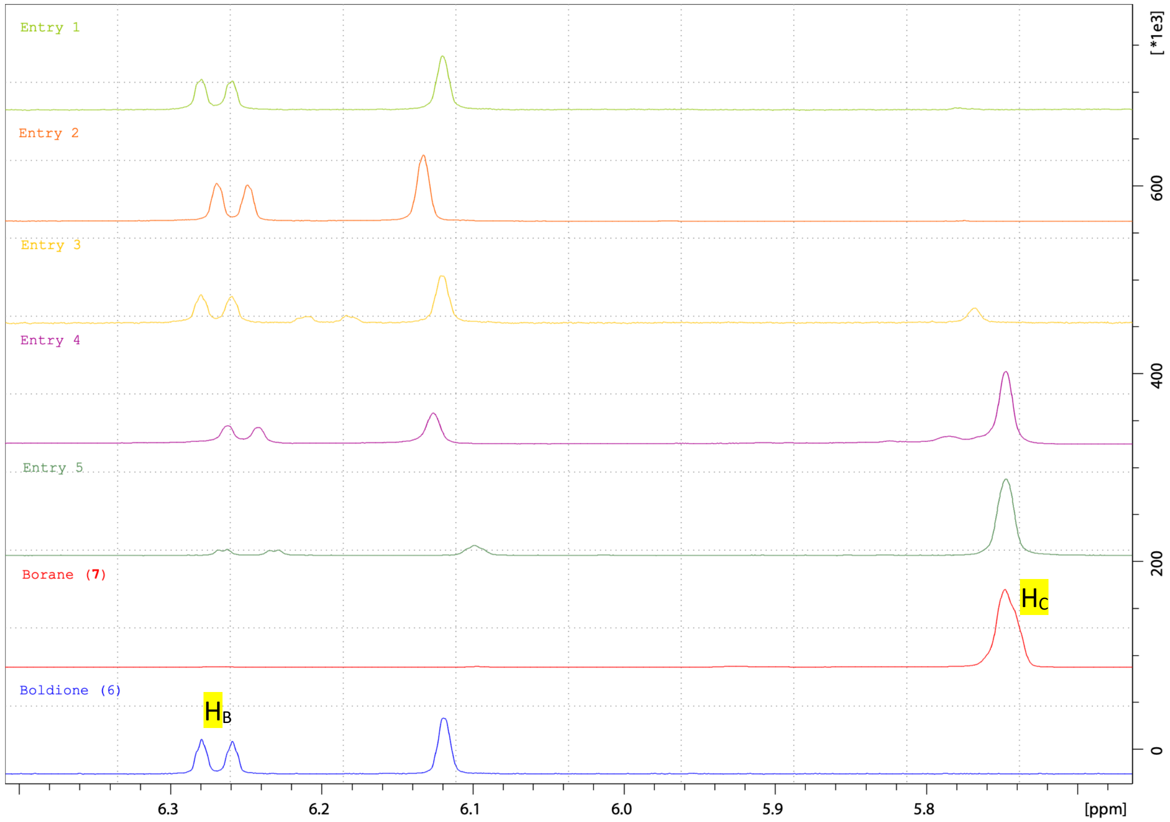

Figure S2-2. (A) Scheme of borylation of boldione (**6**) to give **7** (Step 1). (B) Overlay of Entries 1-5 of the optimization of the borylation reaction from Table S2-1. H_C (position-4, δ 5.8) signal of **7** is most distinct in Entry 5. Boldione (**6**) is on the bottom row (blue) and the borane adduct (**7**) is one row above (red).

3. Optimization of Step 6: Stereoselective Reduction of C1-ketone (Table 2 from Main Text)

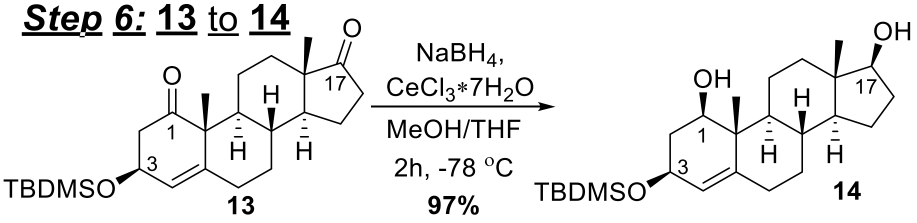

Figure S3-1. Optimization of the Stereoselective Reduction of the C1-Ketone to yield the 1β-Hydroxy Epimer (**13** to **14**, Step 6) (Table S3-1).

Background to the stereoselective reduction of a C1-ketone to yield a 1β-hydroxy group:

Prior efforts to synthesizing a steroid bearing a 1β-hydroxy group involved the epoxidation of the 3-keto-Δ^1 steroid followed by reduction to open the epoxide to yield the 1β-hydroxy group.[4] Although epoxidation and reduction to introduce the 1β-hydroxy group was a previously published strategy, we decided to undertake a different route to obtain the desired product because epoxidation of the 1,2-olefin on a steroid system lacking the 4,5-olefin would require the introduction of the double bond at C4.[4] Therefore, a potential stereoselective reduction of a C1-ketone intermediate bearing a 4,5-olefin would be ideal similar to how our research laboratory previously reported a stereoselective reduction of a C12-ketone intermediate.[5,6] Based on exploring various reduction conditions, it was found that the use of a small hydride such as $LiAlH_4$ (but not L-Selectride) in THF at low temperature (-78 °C) would avoid overcoming the torsional strain and yield the desired 1β-hydroxy stereochemistry.

4. P.K. Sharma, A. Akhila, *Indian J. Chem.* 1991**,** *30B*, 554-556.

5. S.D. Offei, H.D. Arman, M.O. Baig, L.S. Chavez, C.A. Paladini, F.K. Yoshimoto, *Steroids*, 2018, 185-195.

6. S.D. Offei, H.D. Arman, F.K. Yoshimoto, *Org. Biomol. Chem.*, 2023, 21, 3172-3176.

3. Optimization of Step 6: Stereoselective Reduction of C1-ketone (Table 2 from Main Text)

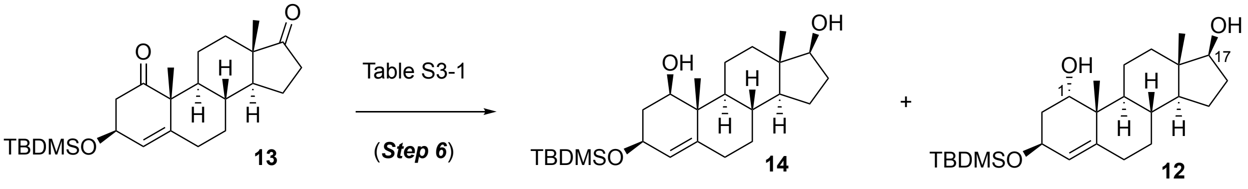

Table S3-1. Stereoselective reduction of a 1,17-diketone steroid (**13**) to yield **14** or **12**. (Step 6)

Entry	Conditions	yield of **14**	yield of **12**
1[a]	L-Selectride (192 eq) in THF (0.2 M), -78 °C	26%	74%
2[a]	$NaBH_4$ (10 eq), CH_3OH (0.2 M), rt	46%	54%
3[a]	$NaBH_4$ (2.0 eq), $CeCl_3 \cdot 7H_2O$ (2.0 eq), CH_3OH (0.2 M), -78 °C	94%	6.0%
4[b]	$NaBH_4$ (3.0 eq), $CeCl_3 \cdot 7H_2O$ (3.0 eq), CH_3OH, rt	53%	47%

[a]: yields determined by integration of ^{1}H NMR signals

[b]: The ratio of the α- and β-hydroxy epimers (**12** and **14**) were determined by by TLC analysis R_f: 0.634 and 0.846, respectively (1 to 1 ethyl acetate/hexanes, v/v).

3. Optimization of Step 6: Stereoselective Reduction of C1-ketone (Table 2 from Main Text)

(A)

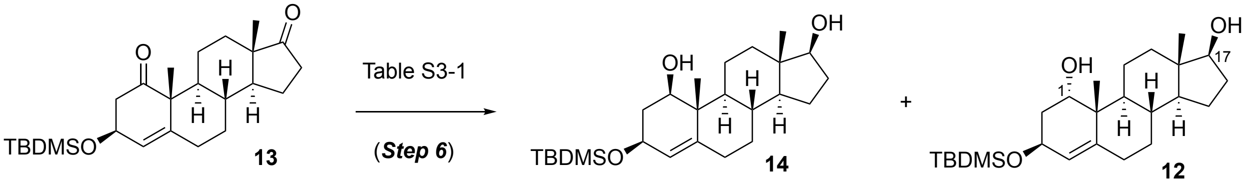

(B)

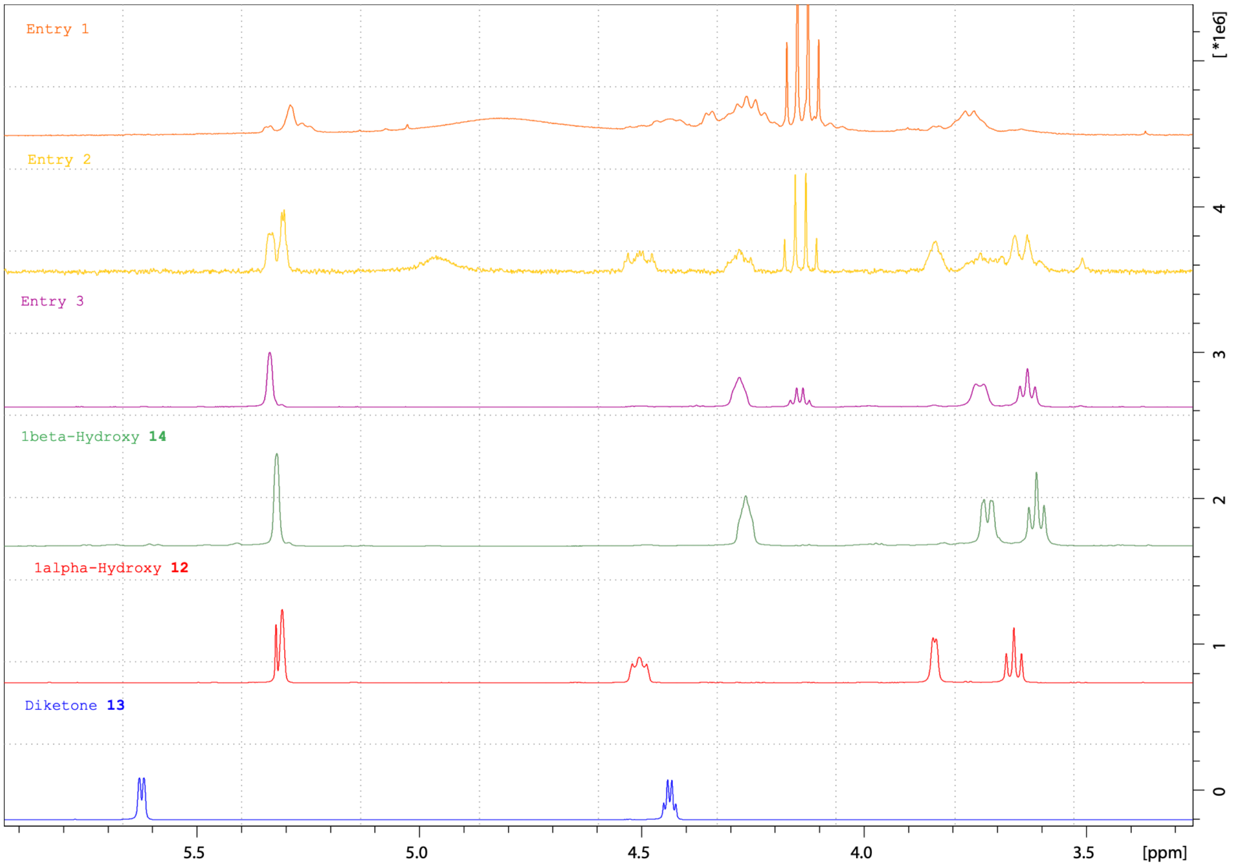

Figure S3-2. (A) Scheme showing the reduction of 1,17-diketone **13** with different conditions shown in Table S3-1 (Step 6). (B) Proton NMR spectroscopic overlay of the different crude reaction mixtures of entries 1-3 (first row at the top to the third row from the top). The bottom row (blue) is the starting material (**13**), the row above (second from the bottom, red) is the 1α-hydroxy epimer **12**, and the third row from the bottom is the desired 1β-hydroxy epimer **14**.

3. Optimization of Step 6: Stereoselective Reduction of C1-ketone (Table 2 from Main Text)

(A)

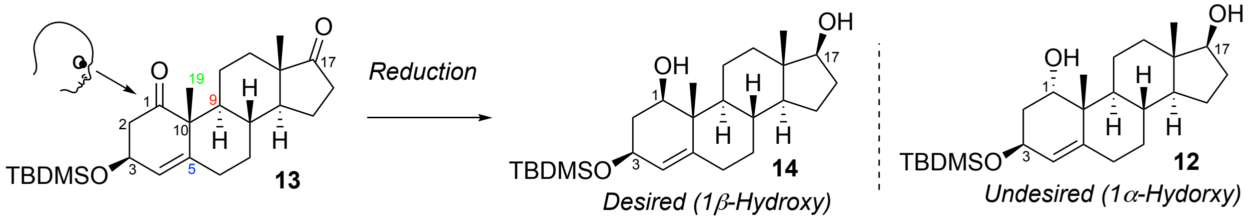

(B)

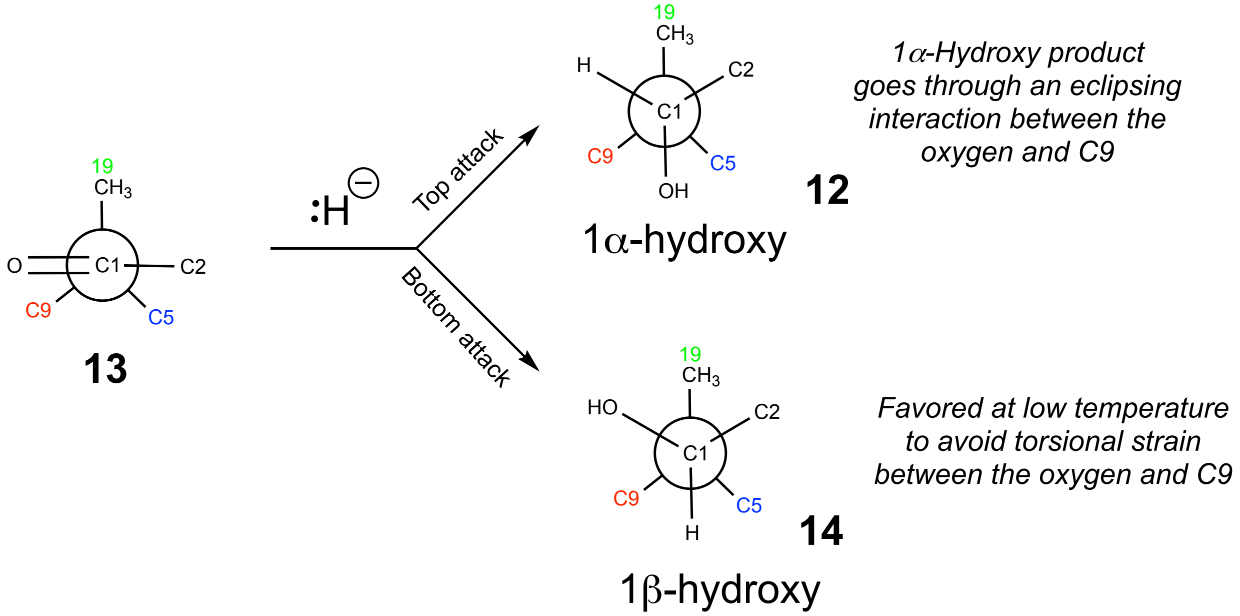

Figure S3-3. (A) Stereoselective reduction of the 1,17-diketone intermediate (**13**) to yield the 1β,17β-diol compound (**14**) was explored (Step 6). **(B)** Newman projection of the C1-C10 dihedral angle showing the axial C19 methyl pointing up in the back. The hydride delivery from the top face undergoes an eclipsing interaction (torsional steering) between the oxygen and the C9 carbon substituent. This torsional strain was overcome when the reaction was performed at room temperature, which led to the undesired 1α-hydroxy product (**12**). However, Luche reduction conditions enabled the reaction to occur at -78 °C and the use of a small hydride reagent, $NaBH_4$, yielding the desired 1β-hydroxy product.

4. X-Ray Structures of Synthesized Compounds

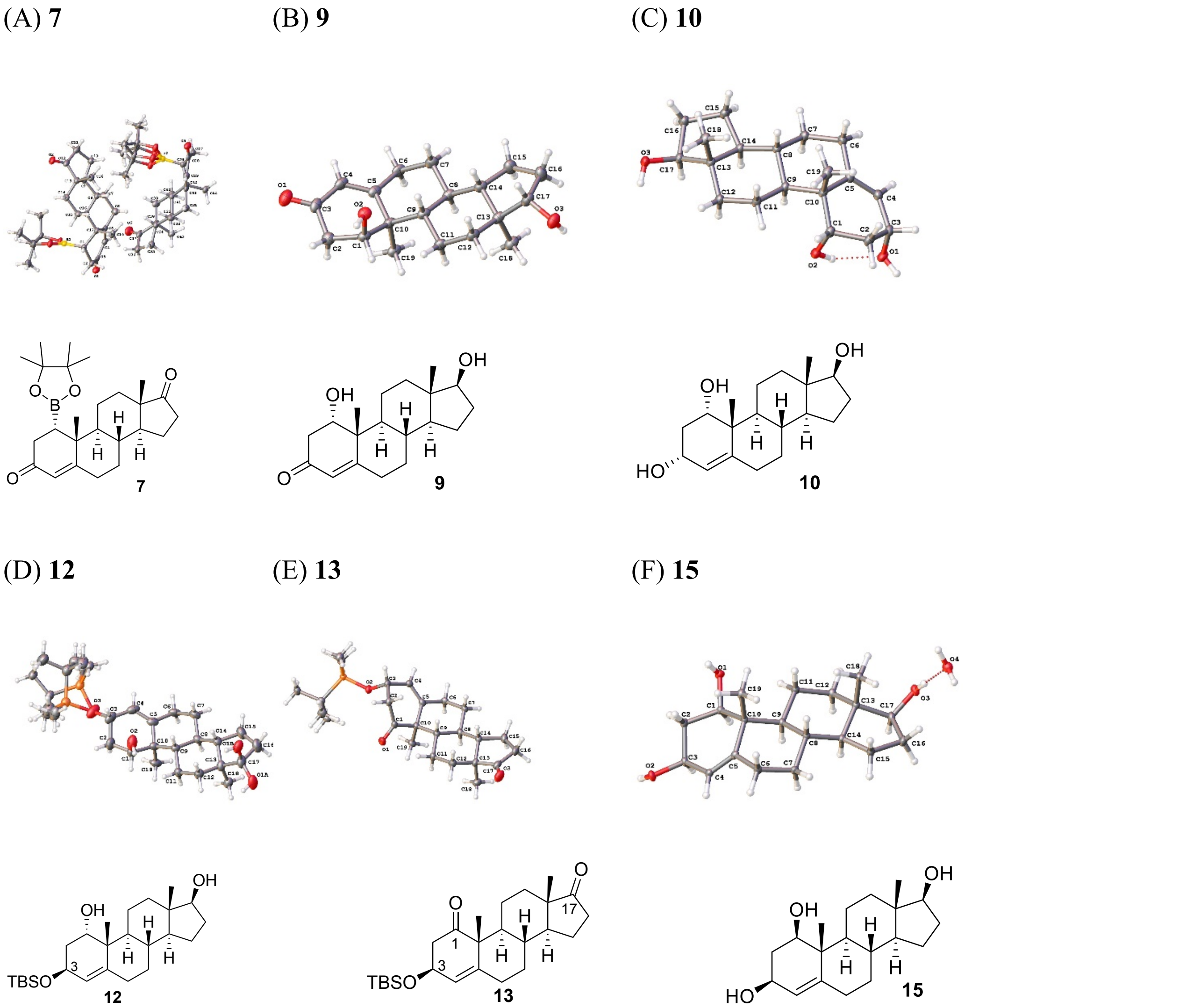

Figure S4-1. Crystal structures of compounds **7**, **9**, **10**, **12**, **13**, and **15**. CCDC deposit numbers for compounds **7**, **9**, **10**, **12**, **13**, and **15** are: 2448105, 2448104, 2448099, 2448100, 2448103, 2448102.

4. X-ray Crystallography of Compounds 7, 9, 10, 12, 13, and 15

Single crystals of $C_{25}H_{37}BO_4$(**7**), $C_{19}H_{28}O_3$ (**9**), $C_{19}H_{30}O_3$ (**10**), $C_{25}H_{43}O_3Si$ (**12**), $C_{25}H_{40}O_3Si$ (**13**), $C_{19}H_{32}O_4$ (**15**), were prepared. For **7** and **13**, the crystals were formed by slow evaporation of a 1:1, ethyl acetate and hexanes (v/v) solution. For **9** and **12**, the crystals were formed by slow evaporation of a 100% ethyl acetate solution. For **10** and **15**, the crystals were formed by slow evaporation of a 100% methanol solution. Suitable colorless plate-like crystals (**7**, **10**, **12**, **13**) and plank-like crystals (**9**, **15**), with dimensions of 0.193 mm × 0.164 mm × 0.058 mm (**7**), 0.153 mm × 0.067 mm × 0.048 mm (**9**), 0.090 mm × 0.063 mm × 0.030 mm (**10**), 0.094 mm × 0.068 mm × 0.052 mm (**12**), 0.227 mm × 0.081 mm × 0.069 mm (**13**), 0.118 mm × 0.112 mm × 0.070 mm (**15**), were mounted in paratone oil onto nylon loops. All data were collected at 100.0(1) K, using a XtaLAB Synergy/ Dualflex, HyPix fitted with CuKα radiation (λ = 1.54184 Å). Data collection and unit cell refinement were performed using *CrysAlisPro* software.[7] The total number of data were measured in the 6.6° < 2θ < 153.5°(7) , 7.4° < 2θ < 140.0°(9), 8.8° < 2θ < 153.2°(10), 5.8° < 2θ < 152.4°(12), 6.2° < 2θ < 153.1°(13), 7.7° < 2θ < 153.2°(15), 5.1° < 2θ < 153.5°(16), using ω scans. Data processing and absorption correction, giving minimum and maximum transmission factors (0.7989, 1.000 (7), 0.8588, 1.000 (9), 0.883, 1.000 (10), 0.4539, 1.000 (12), 0.714, 1.000 (13), 0.683, 1.000 (15), 0.453, 1.000 (16)) were accomplished with *CrysAlisPro*[7] and *SCALE3 ABSPACK*,[8] respectively. The structure, using Olex2,[9] was solved with the ShelXT[10] structure solution program using direct methods and refined (on F^2) with the ShelXL[11] refinement package using full-matrix, least-squares techniques. All non-hydrogen atoms were refined with anisotropic displacement parameters. All hydrogen atom positions were determined by geometry and refined by a riding model. For compound **7**, the boryl group exhibits 80/20 positional disorder in the structure. For compound **12**, the silyl ether group exhibits 70/30 positional disorder and the hydroxyl group, labeled O1, exhibits 75/25 positional disorder in the structure.

7. CrysAlisPro Software System, Version 1.171. 38.41 k, Rigaku Corporation.

8. Oxford Diffraction Ltd. *SCALE3 ABSPACK*; PRO CrysAlis - Yarnton, England. 2009.

9. O. V. Dolomanov, L. J. Bourhis, R. J. Gildea, J. A. K. Howard, H. Puschmann, *J. Appl. Crystallogr.* 2009, *42*, 339.

10. G. M. Sheldrick. SHELXT – Integrated space-group and crystal-structure determination. *Acta Crystallogr., Sect. A: Found. Adv.* 2015, vol. 71, 3-8.

11. G. M. Sheldrick. A short history of SHELX. *Acta Crystallogr., Sect. A: Found. Adv.* 2008, Vol. 64, 112-22.

4. X-ray Crystallography of Compounds 7, 9, 10, 12, 13, and 15

Table S4-1: Crystallographic data and structure refinement for **compounds 7, 9, 10, 12**

Identification code	Compound **7** CCDC: 2448105	Compound **9** CCDC: 2448104	Compound **10** CCDC: 2448099	Compound **12** CCDC: 2448100
Empirical formula	$C_{25}H_{37}BO_4$	$C_{19}H_{28}O_3$	$C_{19}H_{30}O_3$	$C_{25}H_{43}O_3Si$
Formula weight	412.35	304.41	306.43	419.68
Crystal system	Orthorhombic	Monoclinic	Orthorhombic	Orthorhombic
Space group	$P2_12_12_1$	$P2_1$	$P2_12_12_1$	$P2_12_12_1$
a (Å)	11.4856(2)	11.1137(6)	7.59870(10)	7.22960(10)
b (Å)	15.6493(3)	6.2606(2)	12.27340(10)	11.0072(2)
c (Å)	25.3082(5)	13.1134(7)	17.7907(2)	30.5982(5)
α (°)	90	90	90	90
β (°)	90	114.743(6)	90	90
γ (°)	90	90	90	90
Volume (Å^3)	4548.94(15)	828.65(8)	1659.19(3)	2434.93(7)
Z	8	2	4	4
ρ (calc.)	1.204	1.220	1.227	1.145
λ	1.54184	1.54184	1.54184	1.54184
Temp. (K)	100.0(1)	100.0(1)	100.0(1)	100.0(1)
F(000)	1792	332	672	924
μ (mm^{-1})	0.621	0.637	0.636	1.012
T_{min}, T_{max}	0.7989, 1.000	0.8588, 1.000	0.883, 1.000	0.4539, 1.000
$2\theta_{range}$ (°)	6.6 to 153.4	7.4 to 140.0	8.8 to 153.2	5.8 to 152.4
Reflections collected	43168	9081	15752	21739
Independent reflections	9109 [R(int) = 0.0590]	2879 [R(int) = 0.1223]	3350 [R(int) = 0.0353]	4811 [R(int) = 0.0769]
Completeness	97.6%	94.9%	97.4%	96.1%
Data / restraints / parameters	9109 / 0 / 542	2879 / 1 / 207	3350 / 0 / 204	4811 / 0 / 315
Observed data [I > 2σ(I)]	8301	2733	3224	4455
wR(F^2 all data)	0.1512	0.1445	0.0856	0.1370
R(F obsd data)	0.0700	0.0658	0.0314	0.0606
Goodness-of-fit on F^2	1.10	1.10	1.09	1.10
largest diff. peak and hole (e Å^{-3})	0.50 / -0.37	0.28 / -0.28	0.18 / -0.18	0.34 / -0.31

$$wR_2 = \{ \Sigma [w(F_o^2 - F_c^2)^2] / \Sigma [w(F_o^2)^2] \}^{1/2}$$

$$R_1 = \Sigma ||F_o| - |F_c|| / \Sigma |F_o|$$

4. X-ray Crystallography of Compounds 7, 9, 10, 12, 13, and 15

Table S4-2: Crystallographic data and structure refinement for **compounds 13, 15**

Identification code	Compound **13** CCDC: 2448103	Compound **15** CCDC: 2448102
Empirical formula	$C_{25}H_{40}O_3Si$	$C_{19}H_{32}O_4$
Formula weight	416.66	324.44
Crystal system	Monoclinic	Orthorhombic
Space group	$P2_1$	$P2_12_12_1$
a (Å)	11.3196(5)	7.94675(7)
b (Å)	7.2773(2)	9.36032(10)
c (Å)	15.2503(6)	23.0080(3)
α (°)	90	90
β (°)	110.961(4)	90
γ (°)	90	90
Volume (Å^3)	1173.13(8)	1711.43(3)
Z	2	4
ρ (calc.)	1.18	1.259
λ	1.54184	1.54184
Temp. (K)	100.0(1)	100.0(1)
F(000)	456	712
μ (mm^{-1})	1.049	0.688
T_{min}, T_{max}	0.714, 1.000	0.683, 1.000
$2\theta_{range}$ (°)	6.2 to 153.1	7.7 to 153.2
Reflections collected	16148	16382
Independent reflections	3885 [R(int) = 0.0649]	3410 [R(int) = 0.0350]
Completeness	96.3%	96.9%
Data / restraints / parameters	3885 / 1 / 269	3410 / 0 / 222
Observed data [I > 2σ(I)]	3450	3326
wR(F^2 all data)	0.1074	0.0739
R(F obsd data)	0.0424	0.0281
Goodness-of-fit on F^2	1.07	1.03
largest diff. peak and hole (e Å^{-3})	0.38 / -0.22	0.21 / -0.15

$$wR_2 = \{ \Sigma [w(F_o^2 - F_c^2)^2] / \Sigma [w(F_o^2)^2] \}^{1/2}$$

$$R_1 = \Sigma ||F_o| - |F_c|| / \Sigma |F_o|$$

5. References for the SI file

1. H.E. Gottlieb, V. Kotlyar, A. Nudelman, *J. Org. Chem.*, 1997, 62, 7512-7515.

2. B. Pelc, J. Hodkova, *Collect. Czech. Chem. Commun.*, 1967, 32, 410-418.

3. W.R. Benn, F. Colton, R. Pappo, *J. Am. Chem. Soc.*, 1957, 79, 3920.

4. P.K. Sharma, A. Akhila, *Indian J. Chem.* 1991**,** *30B*, 554-556.

5. S.D. Offei, H.D. Arman, M.O. Baig, L.S. Chavez, C.A. Paladini, F.K. Yoshimoto, *Steroids*, 2018, 185-195.

6. S.D. Offei, H.D. Arman, F.K. Yoshimoto, *Org. Biomol. Chem.*, 2023, 21, 3172-3176.

7. CrysAlisPro Software System, Version 1.171. 38.41 k, Rigaku Corporation.

8. Oxford Diffraction Ltd. *SCALE3 ABSPACK*; PRO CrysAlis - Yarnton, England. 2009.

9. O. V. Dolomanov, L. J. Bourhis, R. J. Gildea, J. A. K. Howard, H. Puschmann, *J. Appl. Crystallogr.* 2009, *42*, 339.

10. G. M. Sheldrick. SHELXT – Integrated space-group and crystal-structure determination. *Acta Crystallogr., Sect. A: Found. Adv.* 2015, vol. 71, 3-8.

11. G. M. Sheldrick. A short history of SHELX. *Acta Crystallogr., Sect. A: Found. Adv.* 2008, Vol. 64, 112-22.
